# Mechanisms and energetics of free radical initiated disulfide bond cleavage in model peptides and insulin by mass spectrometry†
†Electronic supplementary information (ESI) available. See DOI: 10.1039/c5sc01305d



**DOI:** 10.1039/c5sc01305d

**Published:** 2015-05-20

**Authors:** Chang Ho Sohn, Jinshan Gao, Daniel A. Thomas, Tae-Young Kim, William A. Goddard III, J. L. Beauchamp

**Affiliations:** a Division of Chemistry and Chemical Engineering , California Institute of Technology , Pasadena , CA 91125 , USA . Email: jlbchamp@caltech.edu; b Materials and Process Simulation Center , Beckman Institute , California Institute of Technology , Pasadena , CA 91125 , USA

## Abstract

We investigate the mechanism of disulfide bond cleavage in gaseous peptide and protein ions initiated by a covalently-attached regiospecific acetyl radical using mass spectrometry (MS). Highly selective S–S bond cleavages with some minor C–S bond cleavages are observed by a single step of collisional activation. We show that even multiple disulfide bonds in intact bovine insulin are fragmented in the MS2 stage, releasing the A- and B-chains with a high yield, which has been challenging to achieve by other ion activation methods. Yet, regardless of the previous reaction mechanism studies, it has remained unclear why (1) disulfide bond cleavage is preferred to peptide backbone fragmentation, and why (2) the S–S bond that requires the higher activation energy conjectured in previously suggested mechanisms is more prone to be cleaved than the C–S bond by hydrogen-deficient radicals. To probe the mechanism of these processes, model peptides possessing deuterated β-carbon(s) at the disulfide bond are employed. It is suggested that the favored pathway of S–S bond cleavage is triggered by direct acetyl radical attack at sulfur with concomitant cleavage of the S–S bond (S_H_2). The activation energy for this process is substantially lower by ∼9–10 kcal mol^–1^ than those of peptide backbone cleavage processes determined by density functional quantum chemical calculations. Minor reaction pathways are initiated by hydrogen abstraction from the α-carbon or the β-carbon of a disulfide, followed by β-cleavages yielding C–S or S–S bond scissions. The current mechanistic findings should be generally applicable to other radical-driven disulfide bond cleavages with different radical species such as the benzyl and methyl pyridyl radicals.

## Abstract 2

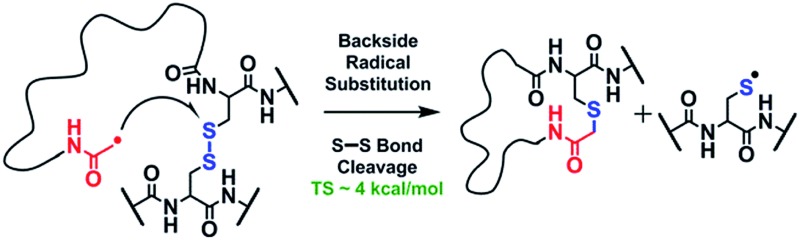
Direct radical substitution at sulfur initiates disulfide bond cleavage by hydrogen-deficient radicals in peptides and proteins.

## Introduction

As an important post-translational modification, identification and characterization of disulfide bonds in proteins are critical for determination of their three-dimensional structure.^1^ The disulfide bond, a strong covalent linking of two protein segments containing cysteine residues, significantly contributes to the stabilization of tertiary structures^2,3^ and helps to maintain protein activity in the cellular environment.^4–7^ Despite their important roles in biological systems, analysis of disulfide bonds in proteins remains a challenging task exacerbated by their fragility toward redox stress. The native disulfide connectivity can be easily lost by reduction and re-oxidation of disulfides, which may occur randomly during sample isolation and preparation for analysis. To avoid this problem, pre-treatment of disulfides by reduction/alkylation or oxidation is often performed.^8–10^ Although these approaches allow sequencing of peptide segments that were previously inaccessible due to disulfide loops, the methods lead to concomitant loss of information related to the structural constraints imposed by disulfide linkages.

Not surprisingly, the rapid expansion of experimental methodology employing high performance mass spectrometry (MS) has included the development of new approaches for disulfide bond characterization.^11^ Recently, top-down mass spectrometry was employed to investigate intact disulfide-bonded protein ions.^12–14^

In common approaches to disulfide bond analysis using MS, proteins of interest are usually subject to protease digestion. Protein digests that retain intact disulfide bonds produced by pepsin typically contain both inter- and intramolecular disulfide linkages. After ionization of protein digests, cleavage of intermolecular disulfide bonds by ion activation leads to separated peptide fragments, and further activation can yield fragments revealing the point of connection. For the case of intramolecular disulfide bonds, it requires multiple steps of activation to locate the linkage sites. Low-energy collision induced dissociation (CID) of protonated peptides containing disulfide bonds usually leads to a mixture of amide backbone and disulfide C–S bond cleavage, with essentially no S–S bond rupture due to the higher activation energy required for this process.^15^ Therefore, only limited structural information can be acquired by conventional low-energy CID of protonated peptides containing intramolecular disulfide bonds.^16^ Some of the low-energy CID approaches with certain limited conditions generate more information-rich fragments. Gaseous peptide ions lacking mobile protons typically exhibit highly selective C–S bond cleavages by low-energy CID.^17^ This effect is especially prominent in singly protonated disulfide containing peptide ions produced by matrix-assisted laser desorption ionization (MALDI).^18^ CID of anionic disulfide bridged peptides also generates cleavage products from C–S bond fragmentation but their intensities are usually weak and the fragmentation pattern is complex.^19–21^

Metal cationized disulfide containing peptides have also been thoroughly investigated by MS. The patterns of disulfide fragmentation with various metal complexes are diverse.^22^ For example, peptides containing disulfide bonds cationized by a gold cation undergo efficient S–S bond cleavages by low-energy CID.^23^ In contrast, alkali or alkaline earth metal–peptide complexes cleave C–S bonds, yielding highly selective H_2_S_2_ loss.^24,25^ This signature neutral loss can be used for fast screening of disulfide containing peptides resulting from peptic digestion. The observed processes are triggered by anionic enolation of cysteine residues at backbone C_α_ positions by metal cations, followed by sequential cleavage of the C–S bonds.

Electron-based dissociations such as electron capture dissociation (ECD)^26,27^ and its variations, electron transfer dissociation (ETD),^28–31^ electron detachment dissociation (EDD),^32,33^ and negative ion electron capture dissociation (niECD),^34^ have proven to be very attractive methods for analysis of disulfide linkages, deriving advantage from selective cleavage of S–S bonds in peptides and proteins. The detailed processes for initial electron capture and subsequent S–S bond cleavage in various disulfide bond containing peptides and proteins remain an active subject for further experimental and theoretical investigations.^35^ Notably, a recent paper raised a concern on less effective disulfide bond cleavage by ECD.^36^

Ultraviolet photodissociation (UVPD) at 157, 193, and 266 nm also produces highly selective disulfide bond cleavages.^37–39^ Homolytic cleavage of S–S bonds was suggested as a mechanism of UVPD of disulfide-linked proteins. However, the requirement for specialized instrumentation hinders wide applications of UVPD in disulfide bond analyses in peptides and proteins.

**Scheme 1:**
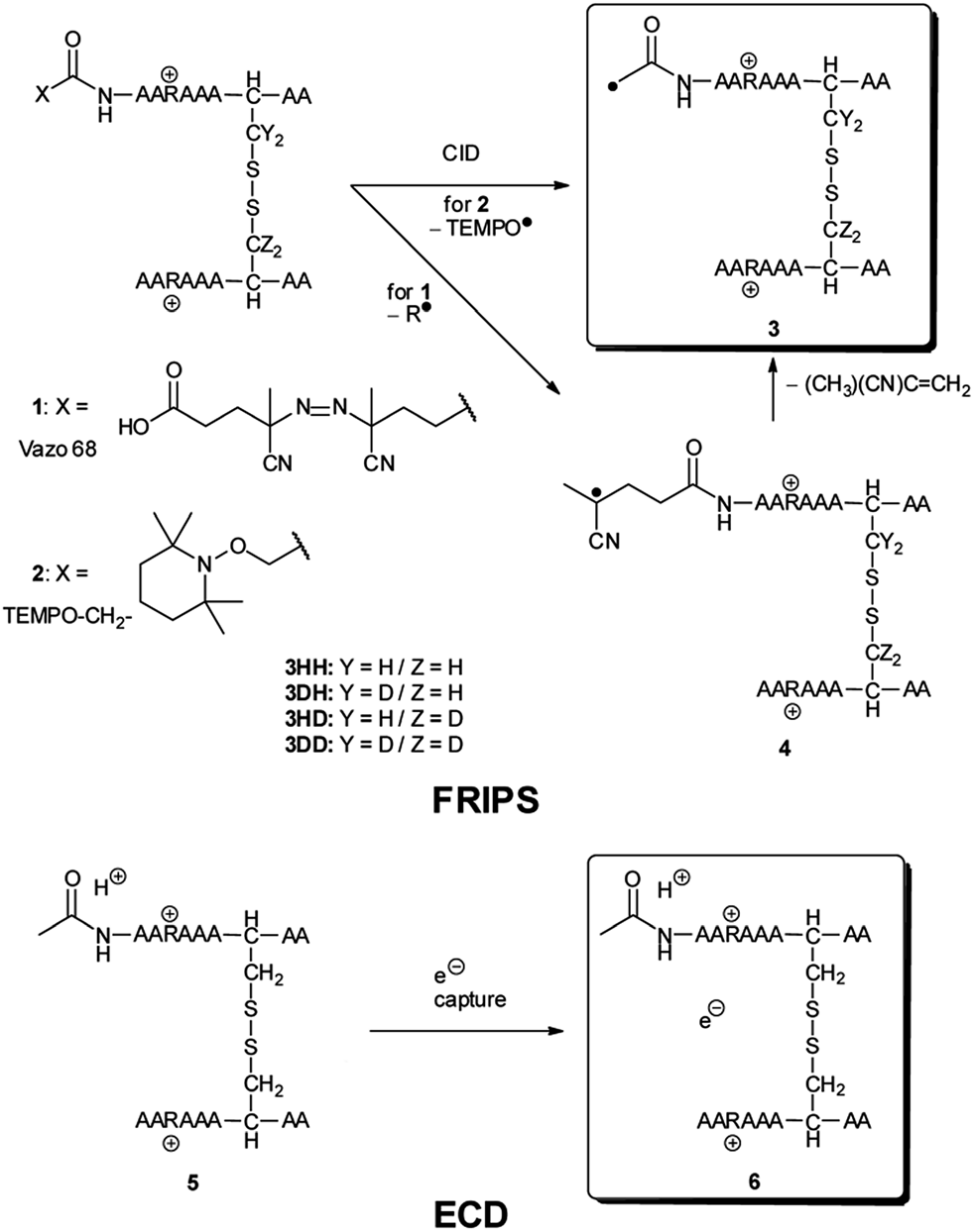


We have previously described an alternative ion activation method, free radical initiated peptide sequencing (FRIPS) *via* multistep collisional activation of peptides conjugated with a reagent (Vazo 68, Scheme 1) that introduces a regiospecific free radical center.^40^ In the present study, we employ a second generation 2,2,6,6-tetramethylpiperidine-1-oxyl (TEMPO)-based FRIPS reagent initially inspired by Lee *et al.* ^41^ that has also been applied in recent studies in our research group.^42,43^ As shown in Scheme 1, TEMPO-based FRIPS reagent peptide conjugates can introduce an acetyl radical group at the peptide N-terminus in a single step of collisional activation.

**Scheme 2:**
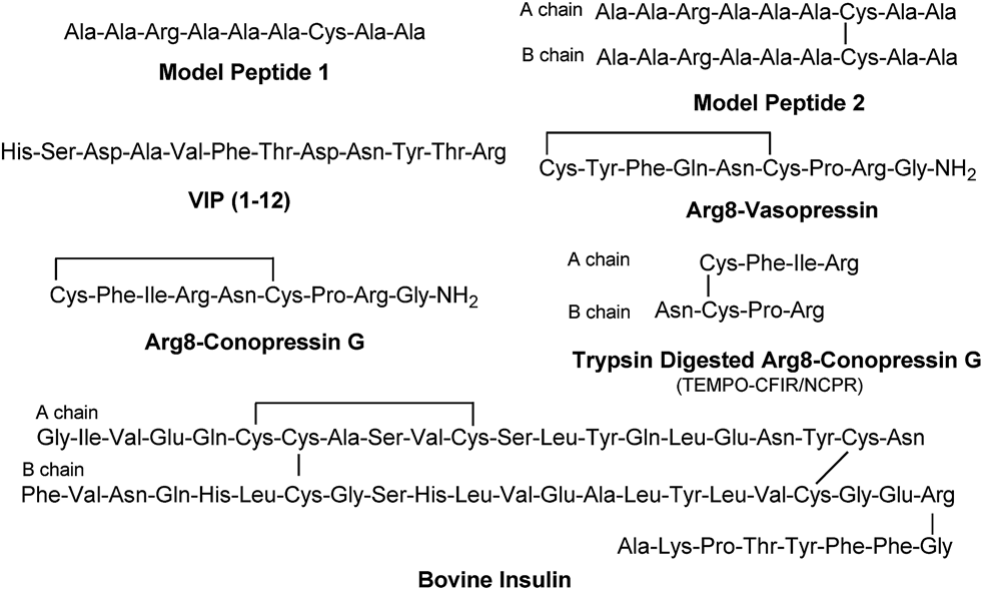


The sequencing performance of this reagent is validated with a set of model systems including the tryptic peptide HSDAVFTDNYTR (Fig. S3, ESI†), the intramolecular disulfide bond containing peptides Arg8-Vasopressin and Arg8-Conopressin G (Fig. S5, ESI†), the intermolecular disulfide bond containing peptide from a tryptic digest of Arg8-Conopressin G, and intact bovine insulin containing one intra- and two interchain disulfide bonds, the latter linking the A- and B-chains together (Scheme 2). Intact bovine insulin is employed to investigate the application of our FRIPS reagent to top-down disulfide analysis.^14^ All model systems used in this study are shown in Scheme 2.

During the course of our research, several other publications appeared relating to the gas-phase free radical cleavage of disulfide bonds.^29,44–46^ Yet, it has remained unclear why (1) disulfide bond cleavage is preferred to backbone fragmentation, and why (2) the S–S bond that requires the higher activation energy conjectured in previously suggested mechanisms is more prone to be cleaved than the C–S bond by hydrogen-deficient radicals. To more thoroughly probe the mechanisms of disulfide bond cleavages by an acetyl radical, model peptides having β-deuteriums at the disulfide bond are employed (Scheme 1, **2** with no deuterium (**2HH**), β-deuteriums at the A-chain (**2DH**), at the B-chain (**2HD**), and at both chains (**2DD**)). Quantum chemical calculations using third generation meta-hybrid density functionals (BMK,^47^ M05-2X,^48^ and M06-2X,^49^ chosen for their better performance in organic radical reactions) along with the conventional B3LYP^50,51^ functional were performed to quantify energetics of observed reaction processes and their proposed mechanistic pathways.

## Experimental section

Details relating to the synthesis of TEMPO-based FRIPS reagent-labeled peptides, mass spectrometry, and computational methods can be found in ESI.† Briefly, the TEMPO-based FRIPS reagent (*N*-hydroxysuccinimide ester) was conjugated to peptides under phosphate buffer at pH 8.5, and the resulting products were desalted and directly infused into LCQ Deca XP and LTQ ion traps or LTQ-FT mass spectrometers for analyses.

## Results and discussion

### Arg8-Vasopressin

**Fig. 1:**
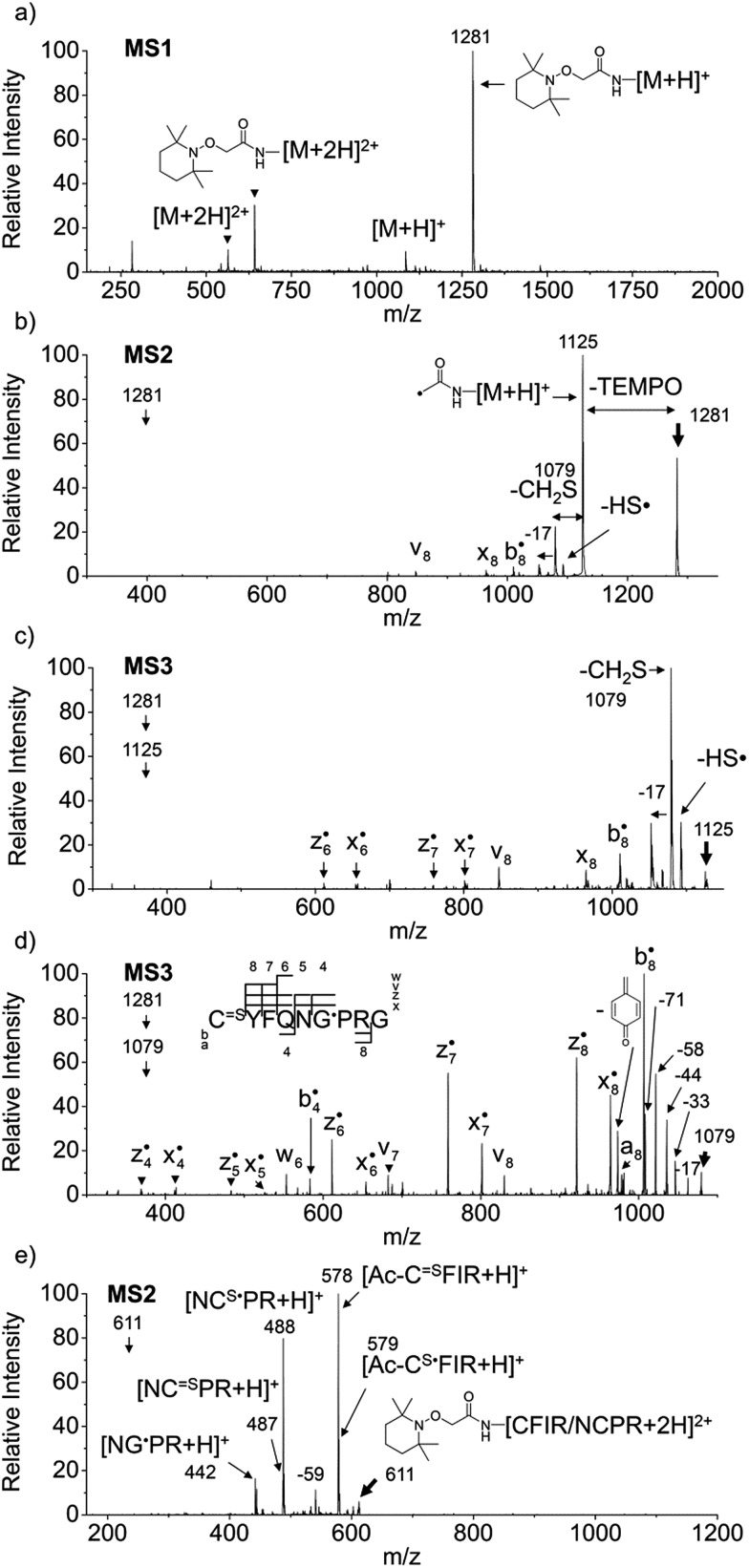
FRIPS of Arg8-Vasopressin and trypsin digest of Arg-Conopressin G. (a) Electrospray ionization (ESI)-MS1 of the TEMPO-based FRIPS reagent conjugate of Arg8-Vasopressin. (b) CID of the singly protonated TEMPO-based FRIPS reagent conjugate of Arg8-Vasopressin, *m*/*z* 1281 (MS2). (c) CID of the acetyl radical cation, *m*/*z* 1125 (MS3). (d) CID of the CH_2_S loss product from the acetyl radical cation, *m*/*z* 1079 (MS3). (e) CID of doubly protonated TEMPO-CFIR/NCPR at *m*/*z* 611 (MS2). C^=S^ is thioaldehyde, thiomorpholin-3-one or thiirane products, and G˙ is glycyl α-carbon radical. See Scheme 3 for the proposed reaction mechanisms. Bold arrows indicate the precursor ions.

Fig. 1a–d depict FRIPS of Arg8-Vasopressin. The TEMPO-based FRIPS reagent was conjugated to the N-terminal amine of Arg8-Vasopressin with a conversion yield of approximately 90% based on the relative signal intensities between FRIPS reagent conjugated and unmodified Arg8-Vasopressin peaks in Fig. 1a. The singly protonated TEMPO-based FRIPS reagent conjugate of Arg8-Vasopressin (*m*/*z* 1281) is collisionally activated to generate the regiospecific acetyl radical cation (*m*/*z* 1125) by loss of TEMPO radical (Fig. 1b). This process is energetically favored to produce the acetyl radical cation in sufficient yield to permit further CID experiments up to MS4 for peptide sequencing. This is less practical when Vazo 68 is used, with the consequence that MS5 is required to characterize the intramolecular disulfide bond in Arg8-Vasopressin.

**Scheme 3:**
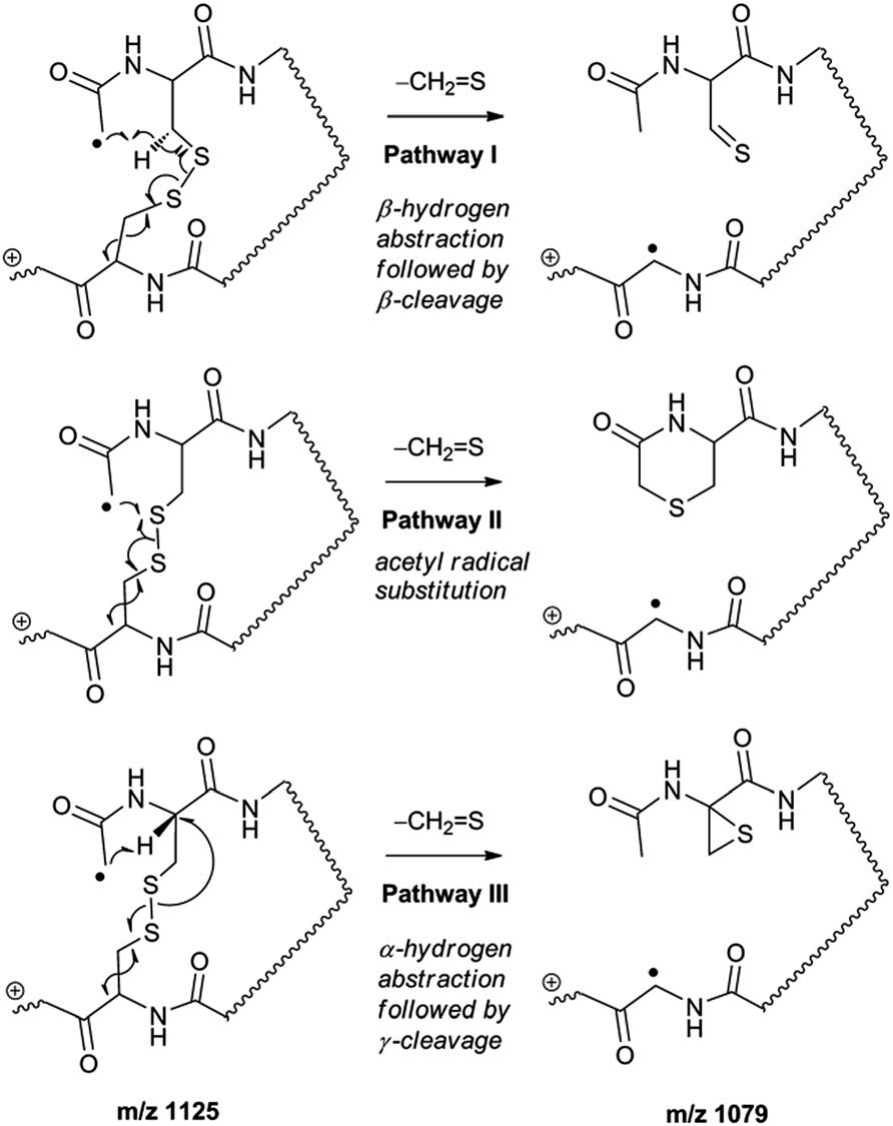


Collisional activation of the acetyl radical cation (*m*/*z* 1125) induces mainly CH_2_S loss (*m*/*z* 1079) by cleaving the S–S bond (Fig. 1c). This process was previously suggested to be initiated by H-atom abstraction at the β-carbon of Cys1, followed by β-cleavage (Scheme 3, pathway I).^44^ The resulting radical cation at *m*/*z* 1079 contains a modified residue whose side-chain is thioaldehyde (–CH=S) at Cys1 position (the 2-amino-3-thioxopropanoic acid residue) and the glycyl α-carbon radical residue at Cys6 position. The possibility of H-atom abstraction at the β-carbon of Cys6 was considered, but no correlated fragments were observed in CID of *m*/*z* 1079 (Fig. 1d). Instead, the six membered ring intermediate favors reaction at the β-carbon of Cys1. No direct β-cleavage from the glycyl α-carbon radical residue (*e.g.* b˙6/y_4_ and b_7_/y˙3) is observed.

Note that unlike the previous FRIPS study by an *o*-benzyl radical,^44^ CH_2_S loss is already prominent in MS2 (Fig. 1b) due to the higher reactivity (*i.e.* higher C–H BDE) of the nascent acetyl radical formed by TEMPO loss.^52^ The glycyl α-carbon radical cation at *m*/*z* 1079 is directly isolated from the MS2 stage and further collisionally activated in MS3 (Fig. 1d).

Subsequent H-atom abstraction by the glycyl radical at other α- or β-carbon sites leads to side-chain losses (17, 33, 58, and 71 Da initiated at the α-carbons) or backbone fragmentation (b, x, z, v and w ions initiated at the β-carbons) by β-cleavage.^53^ From these product ions, the peptide sequence and the position of the intramolecular disulfide bond are assigned (Fig. 1d). Compared to the previous study of alkali and alkaline earth metal complexes of disulfide bond containing peptides,^24^ the sequence coverage after CH_2_S loss is extensive, including 6 out of 8 possible backbone fragments (Fig. 1d).

An alternative mechanism for CH_2_S loss *via* acetyl radical substitution (S_H_2) reaction at the disulfide bond is described in Scheme 3, pathway II.^54^ Radical substitution forms the stable six-membered thiomorpholin-3-one ring structure at the N-terminus, and releases the thiyl radical group by cleaving the S–S bond. The residual internal energy after S–S bond cleavage leads to subsequent loss of CH_2_S, yielding the glycyl α-carbon radical group at Cys6.

H-abstraction at the α-carbon of Cys1, followed by γ-cleavage is also considered (Scheme 3, pathway III). The first step of this pathway, H-abstraction reaction at the α-carbon is energetically favored compared to H-abstraction at the β-carbon.^55^ Also, the final thiirane product is more stable than thioaldehyde, yielding a thermodynamically favored process. However, in general 1,4-H transfer is rarely observed,^56^ and its geometrically imposed energetic constraint compared to 1,5-H transfer renders this pathway kinetically less favored. Note that the overall fragmentation results *via* pathways II and III after loss of CH_2_S are indistinguishable by their mass-to-charge ratios from those of pathway I. In this regard, it is challenging to discern the relative contributions of each reaction pathway proposed in Scheme 3. Differentiation of these mechanisms is accomplished with intermolecular disulfide bond containing peptides that may experience less steric hindrance for H-abstraction at the α-carbon by a more distant radical center instead of the constrained 1,4 interaction, as discussed below.

### Arg8-Conopressin G

FRIPS spectra of doubly protonated Arg8-Conopressin G are shown in Fig. S5 (ESI†). Concomitant losses of TEMPO radical and CH_2_S occur regardless of the charge state (+1 or +2) of the precursor ions in both our model intramolecular disulfide bond containing peptides (Fig. 1 and S5†).

The reactivity of the intermolecular disulfide bond is investigated by collisional activation of doubly protonated TEMPO-CFIR/NCPR (a tryptic digest of the TEMPO-conjugated Arg8-Conopressin G, Fig. 1e). This model system simulates tryptic digests of disulfide bond containing proteins where cleavage fragments in part comprise two peptide chains derived from the original protein backbone, held by an intermolecular disulfide bond. Collisional activation of doubly protonated TEMPO-CFIR/NCPR mainly yields products from S–S bond cleavage. Interestingly, the acetyl radical product from TEMPO loss (–156 Da) is not observed (Fig. 1e). It is believed that most of the nascent acetyl radicals react rapidly to cleave S–S bonds. Rather, loss of 141 Da (2,2,6,6-tetramethylpiperidine) is observed at *m*/*z* 540.8, indicating N–O bond cleavage (Fig. 1e). This product may result from proton transfer from the protonated arginine residue to the TEMPO nitroxide tertiary amine residue and subsequent rearrangement for bond cleavage.

The products resulting from S–S bond cleavage have the thiyl radical and the counterpart even electron species, thioaldehyde, thiomorpholin-3-one or thiirane products, respectively (Scheme 3 and Fig. 1e). Further collisional activation of the products elucidates the site of S–S bond connection with full sequences (Fig. S6, ESI†).

### Intact bovine insulin

**Fig. 2:**
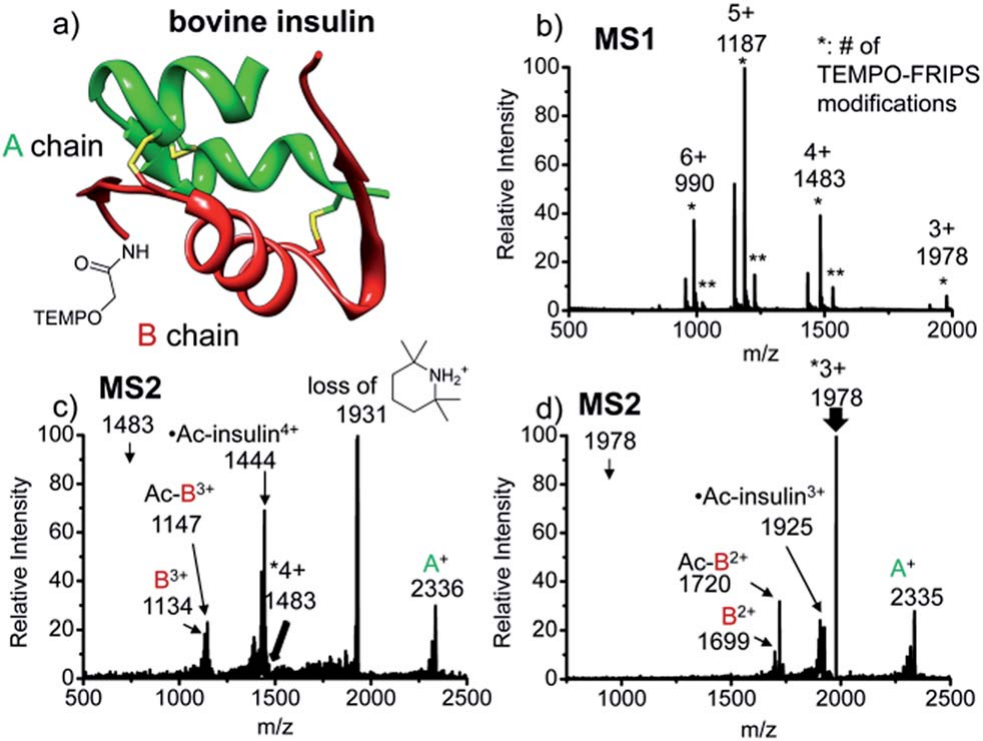
FRIPS of intact bovine insulin. (a) Ribbon modeling of bovine insulin conjugated with FRIPS reagent. (b) ESI-MS spectrum of the TEMPO-derivatized insulin. * denotes the number of TEMPO-FRIPS modifications. (c) FRIPS of singly TEMPO-derivatized 4+ insulin at *m*/*z* 1483. (d) FRIPS of singly TEMPO-derivatized 3+ insulin at *m*/*z* 1978. Highly selective tandem disulfide bond cleavages are observed in 3+ and 4+ insulin ions, releasing A- and B-chain fragments.

FRIPS of intact bovine insulin having not only multiple but also both inter- and intrachain disulfide bonds is shown in Fig. 2. Insulin is conjugated with TEMPO-based FRIPS reagent preferentially at the N-terminus of the B-chain at pH 6.3 to avoid lysine modification and disulfide bond scrambling. Fig. 2b shows that the conjugation of the TEMPO-based FRIPS reagent is efficient, yielding singly derivatized ions as a major species.

As expected, collisional activation of singly TEMPO-based FRIPS reagent-labeled insulin ions results in highly selective S–S bond cleavages (Fig. 2c and d). The acetyl radical selectively cleaves the two inter-disulfide bonds sequentially during the collisional activation, generating the A- and B-chain ions (4+, A^+^ at *m*/*z* 2336, and B^3+^ at *m*/*z* 1134 and Ac-B^3+^ at *m*/*z* 1147; 3+, A^+^ at *m*/*z* 2335, and B^2+^ at *m*/*z* 1699 and Ac-B^3+^ at *m*/*z* 1720). Note that we provide full assignments in ESI† based on high resolution FT-ICR data.¶
¶We provide more details about identities of insulin A-, B-chains (*i.e.* even *vs.* odd electron species) and unassigned backbone fragments with their analyses in Fig. S7–S9 in ESI. The main text focuses on the discussion about disulfide bond cleavages. It is particularly noteworthy that the yield of the A- and B-chain ions is significantly higher than observed using other means of activation, including low energy CID,^16^ ECD,^26^ ETD,^57^ and UVPD at 157 nm ^38^ and 193 nm ^39^ of insulin. Only the previous work by the McLuckey group using low energy CID of gold(i) cation complexes showed the formation of abundant A- and B-chain products.^58^

The tandem disulfide cleavage observed here may be initiated by acetyl radical addition to the first disulfide bond between the A-chain Cys7 and the B-chain Cys7, followed by recyclization of a nascent thiyl radical at the A-chain Cys7 to the A-chain Cys20, forming the cyclic A-chain the and thiyl radical B-chain by cleaving the second disulfide bond. A similar sequential radical reaction was previously reported for ETD of disulfide containing peptides.^59^ The tandem disulfide cleavage ultimately yields the scaffold structure of bovine insulin. Most of the satellite peaks near the A- and B-chain ions result from various neutral losses. In the MS2 spectra, not many backbone fragmentations occur in the 3+ ion while collisional activation of the 4+ ion produces relatively weak (intensity < 5%) backbone fragments outside the interchain disulfide bond loop (Fig. 2c and, the backbone fragment peak assignment is provided in Fig. S7 and S8†). Subsequent collisional activation of the A- and B-chain ions provides the sequencing information for the A- and B-chains, revealing the points of disulfide bond connections (Fig. S9, ESI†).

### AARAAACAA dimer

**Fig. 3:**
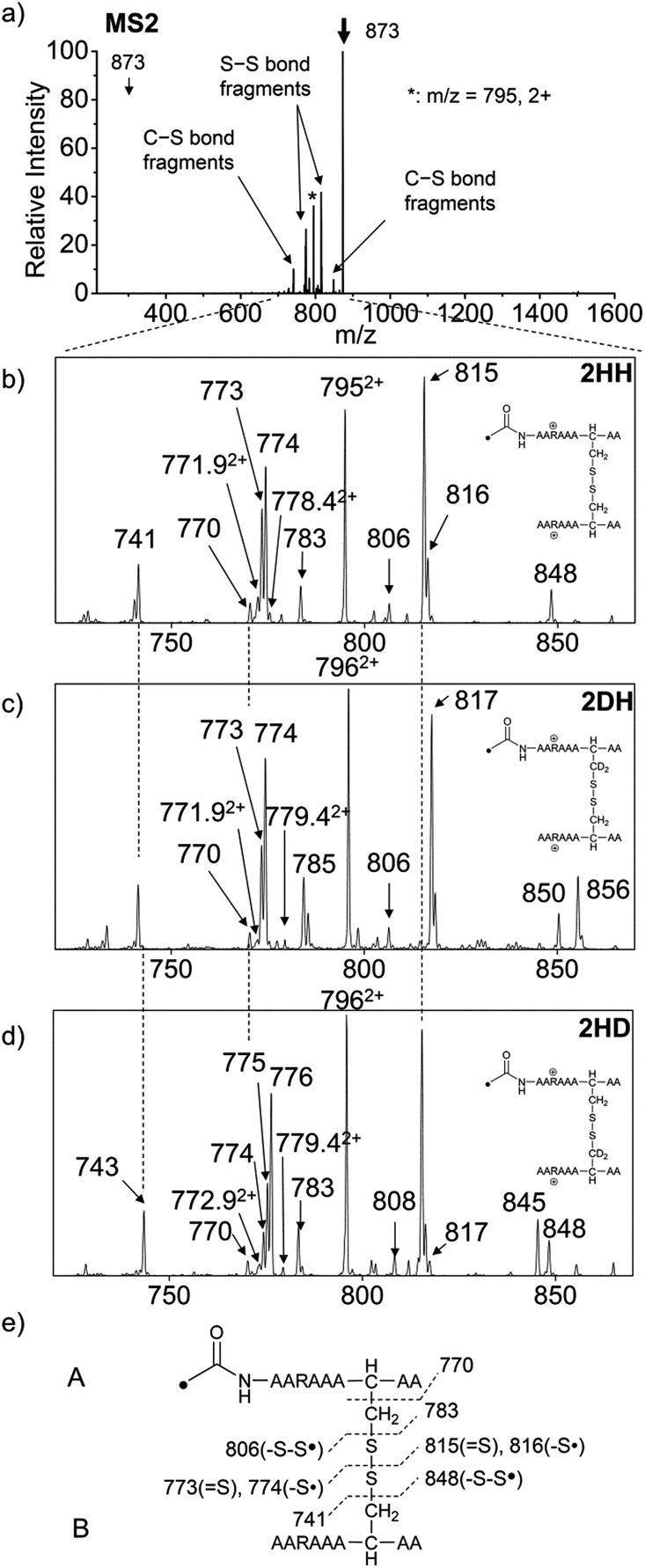
(a) FRIPS of the doubly protonated AARAAACAA disulfide-bridged dimer (**2HH**, *m*/*z* 873, (a and b)) and its deuterated species **2DH** and **2HD**, respectively (c and d). (b–d) Expansion of the *m*/*z* range in which disulfide cleavages occur. (e) Scheme showing cleavage sites and fragment *m*/*z* values from each chain in **3HH**. **3HH** at *m*/*z* 795 in (a) is generated by collisional activation of **2HH** at *m*/*z* 873 *via* loss of TEMPO radical. Essentially no backbone fragmentation is observed. Highly selective C–S (*m*/*z* 741/743, 783/785, 806/808, and 848/850) and S–S cleavage (*m*/*z* 773/775, 774/776, 815/817, 816/818) products are observed. No significant difference is observed in the relative abundances of the products from S–S bond cleavage ([*m*/*z* 817 in **2DH**] *vs.* [*m*/*z* 815 in **2HH**], [*m*/*z* 775 in **2HD**] *vs.* [*m*/*z* 773 in **2HH**]) among FRIPS of **2HH**, **2DH**, and **2HD**.

For the elucidation of the mechanism of observed disulfide bond cleavages, we proceed to a simple model system, a disulfide-linked AARAAACAA dimer. Fig. 3 demonstrates disulfide bond cleavages effected by the acetyl radical in the model system, **3** and its deuterated species. The regioselective acetyl radical dication (*m*/*z* 795) is generated by collisional activation of the doubly protonated AARAAACAA peptide dimer derivatized with the TEMPO-based FRIPS reagent (**2HH**, *m*/*z* 873, Fig. 3a).

In addition, without further collisional activation in MS2, collisional activation of **2HH** dominantly leads to cleavage of the disulfide linkage, yielding various C–S (*m*/*z* 741, 783, 806, and 848) and S–S (*m*/*z* 773, 774, 815, and 816) bond cleavage fragments from each chain (Fig. 3b and e). Table S1† lists theoretical and experimental mass-to-charge ratios and their mass accuracies measured by an ion trap and Fourier transform-ion cyclotron resonance (FT-ICR) MS, respectively (ESI†).

Compared to the FRIPS spectrum of doubly protonated TEMPO-CFIR/NCPR in Fig. 1e, some C–S bond cleavage fragments are observed in Fig. 3. Essentially no backbone fragmentation is observed due to the higher bond dissociation energy of the C_β_–H bond in alanine residues (Fig. 3a).^55^ In addition, S–S bond cleavage is more favored relative to C–S bond cleavage (Table 1). Collisional activation of the acetyl radical dication at *m*/*z* 795 yields numerous fragment ions *via* further losses of HS˙, HSS˙ and CH_2_S (Fig. S10, ESI†). The resulting fragments complicate our analysis on the distribution of C–S and S–S bond cleavages solely produced by an acetyl radical. Therefore, we used MS2 results where further neutral losses are minimized after disulfide bond cleavage, for product distribution comparison (Table 1). ECD of the triply charged intermolecular disulfide containing model peptide (**5**) is performed (Fig. S11, ESI†) for comparison of the reactivity of the nascent charge reduced radical dication to that of the regiospecific acetyl radical dication generated by FRIPS. The charge-reduced model peptide radical dication (**6**) produced by electron capture undergoes both backbone and disulfide fragmentations (Table 1). As noted in the introduction, disulfide bond cleavage is one of the most prominent reaction pathways in ECD and the process has been interpreted according to the viewpoints of both the Cornell^27^ and Utah-Washington^60^ mechanisms. More specifically, even compared to FRIPS of **2HH**, ECD is dominated by S–S bond cleavage, in preference to other C–S bond and backbone fragmentations leading to c and z type ions (Table 1).

**Table 1:** Fragment ions from FRIPS and ECD of AARAAACAA disulfide bridged dimer and their relative yields

Fragment type	Relative yield (%)	
	FRIPS	ECD
Backbone	0.5	15.7
Side-chain loss	1.1	∼0
Overall disulfide	98.4	84.3
C–S bond cleavage	28.0	8.3
S–S bond cleavage	72.0	91.7

### Deuterium labeled AARAAACAA dimer

To further probe the mechanisms of disulfide bond cleavage by an acetyl radical, we introduced β-deuteriums at disulfide bonds of the A- and B-chains in the model peptides (see Scheme 1, **2HH**, **2DH**, **2HD**, and **2DD**, respectively). Comparison of peak intensities indicates the effect of isotopic substitution on product distributions, providing insights relating to the reaction mechanism.

Fig. 3b–d show the FRIPS spectra of **2HH**, **2DH**, and **2HD**, respectively. For C–S bond cleavage, H-abstraction at the α-carbon, followed by β-cleavage may occur, yielding the products at *m*/*z* 741/743, 783/785, 806/808, and 848/850, respectively. It is clear that their relative abundances are almost identical among different deuterium/hydrogen isotopomers. For S–S bond cleavage, if the mechanism involves H-abstraction at the β-carbons, potential kinetic isotope effects on the fragmentation pattern is expected to be observed from these experiments.^61^ However, no significant change is observed in the relative abundances of the products involving S–S bond cleavage ([*m*/*z* 817 in **2DH**] *vs.* [*m*/*z* 815 in **2HD**], Fig. 3). From this result, it is suggested that the mechanism for the formation of the peaks at *m*/*z* 815/817 does not involve H-abstraction from the β-carbons and may instead occur *via* pathways II and III indicated in Scheme 3. If the S–S bond cleavage product at *m*/*z* 815 in FRIPS of **2HD** is formed *via* acetyl radical substitution at the sulfur atom on the A-chain side, a cyclic product between the N-terminal acetyl carbon and the sulfur in the A-chain is generated. Additional collisional dissociation of the cation at *m*/*z* 815 from FRIPS of **2HH** indicates that its dominant form is a cyclic structure, producing internal fragments (Fig. S12, ESI†). However, this cyclic cation has the same mass-to-charge ratio as that produced by H-abstraction at the α-carbon, followed by γ-cleavage (pathway III in Scheme 3), which makes measurement of the contribution of the direct radical substitution mechanism challenging from this experiment.

To further analyze the effect of isotope substitution in the B-chain, the mass-to-charge ratios of the product ions from the B-chain of **2HD** are investigated. By comparing the mass shifts at *m*/*z* 773–776 in the FRIPS spectra of **2HH** and **2HD** (Fig. 3b and d, respectively), the relative contributions of each reaction pathway suggested in Scheme 3 can be clearly ascertained (Table 2). Using Table 2, we can compare the relative product distribution between the pathways. Firstly, based on the peak at *m*/*z* 774 in Fig. 3d, we confirm D-abstraction at the β-carbon followed by β-cleavage as one of the possible pathways (pathway I, Scheme 3).

**Table 2:** Mass-to-charge ratios of the B-chain fragments of **2HD**

Pathway	Hydrogen species	*m*/*z*	Deuterium species	*m*/*z*
I	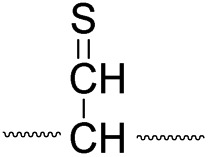	773	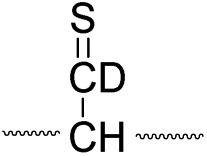	774
II, A-chain	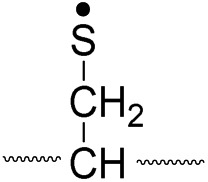	774	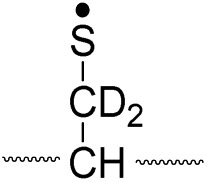	776
II, B-chain	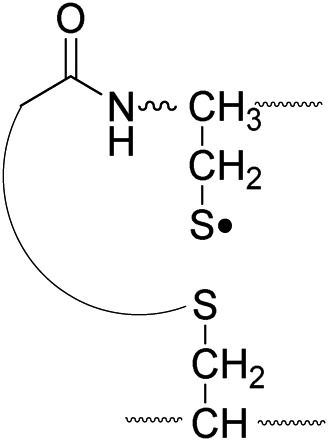	795.4	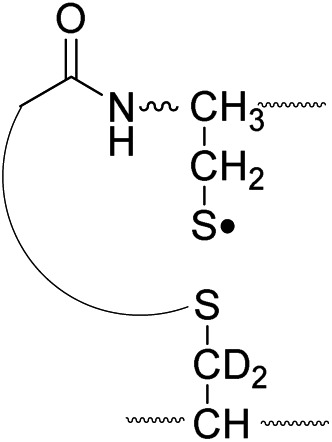	796.4
III	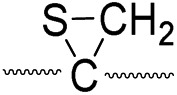	773	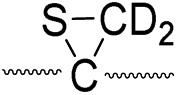	775

Secondly, the peak at *m*/*z* 775 in Fig. 3d can only be explained by the mechanism in which no D-abstraction occurs at the β-carbon (pathway III, Scheme 3). Note that the initial H-abstraction at the α-carbon is not affected by deuterium substitution at the β-carbons. In addition, the final thiirane and thiyl radical products can explain the observed peaks at *m*/*z* 775 and 816 in FRIPS of **2HD** where both deuteriums are still attached to the B-chain product at *m*/*z* 775. Therefore, it is proposed that the process for S–S bond cleavage is also partially initiated by H-abstraction at the α-carbon, followed by γ-cleavage, yielding a thiirane and thiyl radical.

Lastly, the peak at *m*/*z* 776 in Fig. 3d is the thiyl radical ion produced by disulfide cleavage that is not associated with β-carbon deuteriums in the B-chain (pathway II, A-chain in Table 2). Direct association to the β-sulfur position in the B-chain yields intact thiyl radical dications (pathway II, B-chain in Table 2). Subsequent loss of CH_2_S yields the glycyl α-carbon radical as a doubly protonated species (for **2HH** at *m*/*z* 771.9 and for **2HD** at *m*/*z* 772.9). For FRIPS of **2DH**, loss of CD_2_S is observed at *m*/*z* 771.9, supporting pathway II, B-chain in Table 2 where deuteriums are labeled in the A-chain for this case.

Based on the analysis above, we suggest that *S–S bond cleavage can be explained by a combination of all three pathways outlined in* Scheme 3. A significant contribution of pathway II (direct radical substitution) explains both (1) cyclic products at *m*/*z* 815 for **2HH**, **2HD** and at *m*/*z* 817 for **2DH** and (2) dication CH_2_S/CD_2_S loss. Pathway II plays a major role in the formation of even-electron species at the A-chain, while pathway III (H-abstraction at the α-carbon, followed by γ-cleavage) is the dominant process for B-chain even-electron species at *m*/*z* 773 for **2HH**, **2DH** and at *m*/*z* 775 for **2HD**. Pathway I (H-abstraction at the β-carbon, followed by β-cleavage) may play a minor role; the products at *m*/*z* 774 and 817 in FRIPS of **2HD** can be only explained by deuterium abstraction (Fig. 3d). Considering the kinetic isotope effect expected for deuterium abstraction, the actual contribution of pathway I would be more significant for non-deuterated disulfide bond cleavages. For C–S bond cleavage, H-abstraction at the α-carbon may occur, followed by β-cleavage.

### Quantum chemical computations

**Fig. 4:**
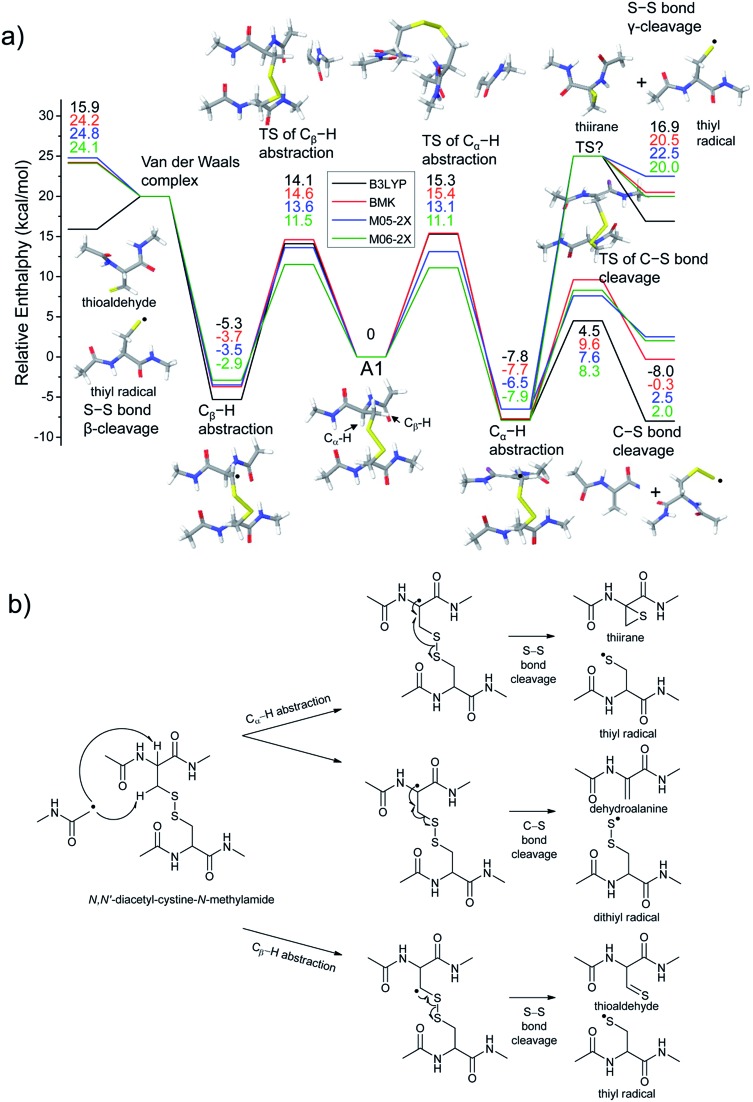
(a) Reaction energetics for S–S bond cleavage (left side) and C–S bond cleavage (right side) of *N*,*N*′-diacetyl-cystine-*N*-methylamide *via* hydrogen abstraction from α- and β-carbons, followed by β- and γ-cleavages showing relative enthalpies in kcal mol^–1^. Geometry optimization and thermochemical calculation (298.15 K and 1 atm) were performed using B3LYP/6-311++G(d,p) level of theory and single point energy refinement was performed using B3LYP (black), BMK (red), M05-2X (blue), and M06-2X (green) density functionals with the 6-311++G(3df,3pd) basis set, respectively. Some barrier heights are not known. *N*-methylacetamide radical (˙CH_2_–CONH–CH_3_) and *N*-methylacetamide are omitted in molecular structure drawings except for transition states of the α- and β-hydrogen abstraction and their enthalpies are included in the relative enthalpy diagram. (b) Schematic drawing of reaction mechanisms for S–S bond cleavage (top and bottom arrows) and C–S bond cleavage (center arrow) of *N*,*N*′-diacetyl-cystine-*N*-methylamide *via* hydrogen abstraction from α- and β-carbons, followed by β- and γ-cleavages.

To investigate the energetics of the observed disulfide cleavage processes in collisionally activated acetyl radical cations, we use *N*,*N*′-diacetyl-cystine-*N*-methylamide and the untethered *N*-methylacetamide radical (˙CH_2_–CONH–CH_3_) as a model system (Fig. 4b). Several low energy conformers of this model system are shown in Fig. S13 (ESI†).

The most stable conformer **A1** is the all-trans form for amide bonds and hydrogen bonds are formed between amide oxygens and *N*-hydrogens in each chain (Fig. 4a). Due to conformational diversity in the model system, we limit our consideration of reaction energetics to the lowest energy structure in each reaction process.

We first investigate C–S and S–S bond cleavages *via* abstraction of hydrogen atoms from α- and β-carbons, followed by β-^40,41^ and γ-cleavages, respectively. Relative enthalpy changes associated with each reaction channel are shown in Fig. 4. For both C–S and S–S bond cleavage reactions, the enthalpy changes predicted by B3LYP systematically deviate from the results estimated by other functionals by ∼8–10 kcal mol^–1^ (Fig. 4a). This systematic deviation by B3LYP in the energetics of organic radical reactions has been reported previously.^47,62,63^ The better performances of BMK and M05/06-2X functionals have been demonstrated in comparison with G3(MP2)-RAD results.^47,62,63^ Therefore, we will discuss the energetics derived from the other three functionals, which are all in reasonable agreement.

As seen in Fig. 4, H-abstraction at the β-carbon is exothermic but is a slightly less favored reaction (∼4 kcal mol^–1^) than H-abstraction at the α-carbon. Barriers for H-abstraction at each carbon are quite similar (∼11–15 kcal mol^–1^). The subsequent β-cleavage reaction of the C–S bond is ∼7–10 kcal mol^–1^ endothermic, yielding acetyl-*N*-methyl dehydroalanine and acetyl-*N*-methyl cysteinyl radical with a ∼14–17 kcal mol^–1^ barrier. The overall enthalpy change for C–S bond cleavage is only ∼0–2 kcal mol^–1^ endothermic. For the process of S–S bond cleavage *via* H-abstraction at the β-carbon, followed by β-cleavage, no conformer of the transition state was found. Instead, it forms a van der Waals complex between thioaldehyde and thiyl radical. For the dissociation of a van der Waals complex, a small barrier needs to be overcome by breaking two hydrogen bonds between amide bonds.

H-abstraction at the β-carbon, followed by S–S bond cleavage is more endothermic by ∼22 kcal mol^–1^ than that of H-abstraction at the α-carbon and subsequent C–S bond cleavage. In the S–S bond cleavage pathway *via* H-abstraction at the α-carbon, followed by γ-cleavage, the overall enthalpy change is ∼2–4 kcal mol^–1^ favored over H-abstraction at the β-carbon, followed by S–S bond cleavage. In this regard, it is shown that the energetics of thiirane formation is more favored than that of thioaldehyde. It is also notable that the transition states for S–S bond scission *via* γ-cleavage may have narrow and tight potential energy surfaces. As a result, it may be much less sampled in the peptide conformation space. The loose transition state for S–S bond scission *via* β-cleavage would be more populated. In summary, it is expected that two mechanisms initiated either by H-abstraction at the β-carbon, followed by β-cleavage (pathway I) or H-abstraction the α-carbon, followed by γ-cleavage (pathway III) may compete with each other for S–S bond cleavage by the interplay of energetics and sampling frequency.

The clear preference for S–S bond cleavage over C–S bond and backbone cleavages observed in all of the experiments described above is not consistent with the computation results summarized in Fig. 4. To provide a reasonable explanation for this important observation, we proceed to quantify other processes by DFT.

**Fig. 5:**
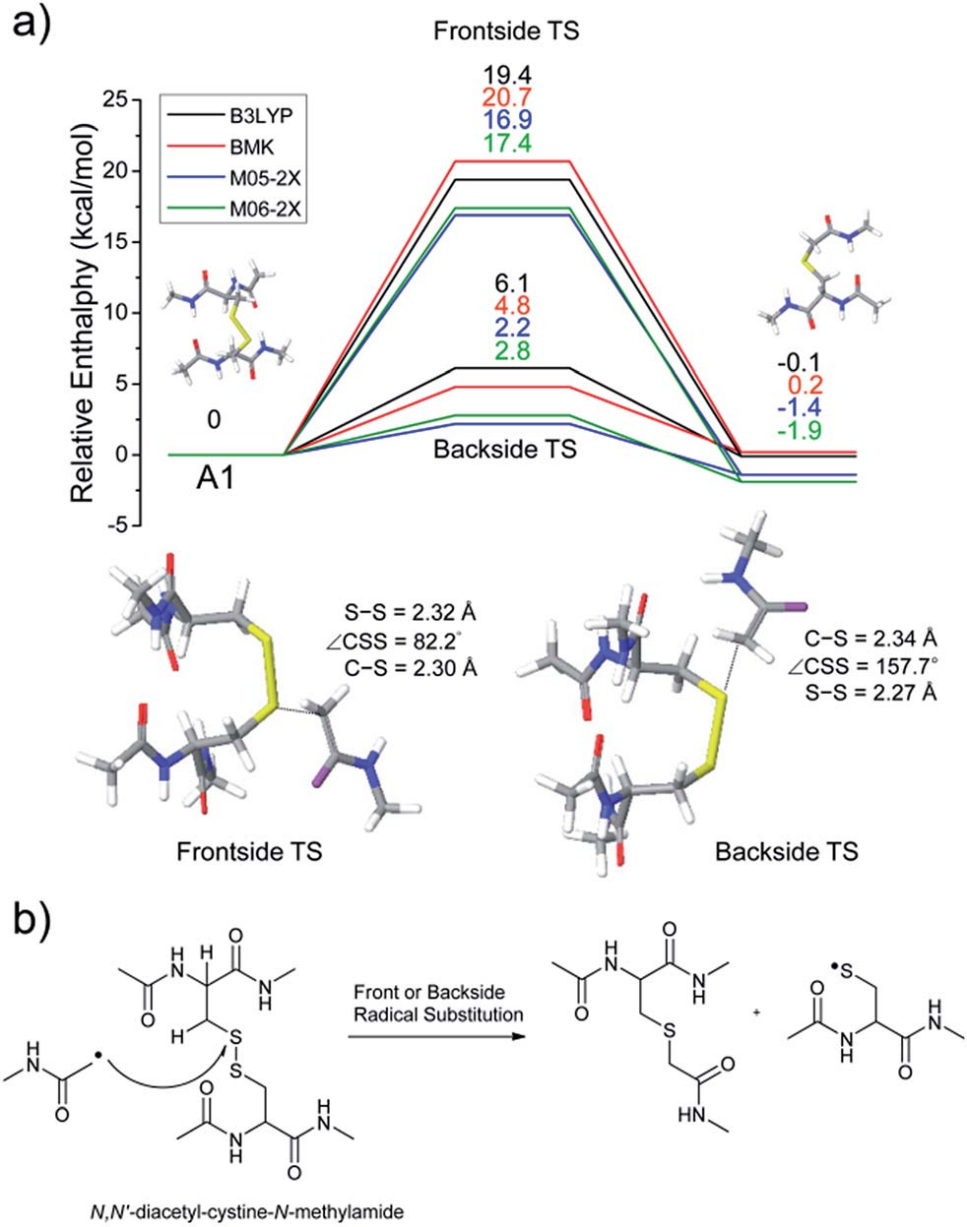
(a) Reaction energetics for S–S bond cleavage of *N*,*N*′-diacetyl-cystine-*N*-methylamide by direct radical substitution *via* front- or backside, showing relative enthalpies in kcal mol^–1^. (b) Schematic drawing of reaction mechanisms for direct radical substitution.

The energetics of the direct acetyl radical substitution to the sulfur atom, followed by S–S bond cleavage is next considered (Fig. 5). Methyl radical substitution to dimethyldisulfide has previously been examined using DFT.^54^ Two distinctive transition states were reported *via* front- and backside attack of the methyl radical and were observed to occur in a concerted process. In the backside attack, the good orbital overlap between the σ* orbital of the S–S bond and the singly occupied molecular orbital (SOMO) of the methyl radical lowers the barrier for S–S bond cleavage.

For the system studied here, the formation of the hypervalent sulfur radical by substitution of the acetyl radical group is investigated to determine whether the process is concerted or possibly involves a stable intermediate. However, intermediate structures having no imaginary vibrational frequency (*i.e.*, non-transition state structures) for the hypervalent sulfur radical were not found. Therefore, concomitant dissociation of an S–S bond by addition of the acetyl radical is predicted to occur by a concerted reaction pathway.

Enthalpy changes for S–S bond cleavage *via* direct addition of the acetyl radical group are estimated to be –0.1, 0.2, –1.4, and –1.9 kcal mol^–1^ by the B3LYP, BMK, M05-2X, and M06-2X/6-311++G(3df,3pd)//B3LYP/6-311++G(d,p) levels of theories, respectively (Fig. 5). The overall process is energetically favored (Δ*H* = ∼0 kcal mol^–1^) compared to the β-hydrogen abstraction initiated process (Δ*H* = ∼24 kcal mol^–1^). Also, the barrier for the backside attack (∼2–5 kcal mol^–1^) is substantially lower than all other reaction pathways including peptide backbone fragmentations. This result also clearly explains dominant disulfide bond cleavages in peptide and protein ions containing disulfide bonds. The nature of radical centers may change the barrier height; nonetheless, it is expected that S–S bond cleavage *via* direct addition of other hydrogen-deficient radicals would be preferred *via* the same pathway if the steric hindrance is not severe. We also compared the similar concept of direct substitution of the acetyl radical for C–S bond cleavage in our computational model. It was found that the barrier of C–S bond cleavage *via* direct radical substitution is substantially higher than that of S–S bond cleavage (backside ∼26–32 kcal mol^–1^, frontside ∼46–51 kcal mol^–1^, Fig. S14, ESI†). This conclusion clearly explains the dominant preference for free radical initiated S–S bond cleavage found in many of the experimental results reported in this work.

Regardless of the significant contribution to S–S bond cleavage of the direct radical addition pathway, it should be noted that the alignment of reactant residues, the acetyl radical and the disulfide bond, is of particular importance for this radical substitution reaction. The reaction barrier is very sensitive to the incident angle of the incoming acetyl radical (frontside *versus* backside, Fig. 5a). It is believed that the conformers where successful orbital overlap occurs between the σ* orbital of the S–S bond and the SOMO of the acetyl radical may not be highly populated due to limited conformation space associated with a low energy reaction coordinate. Therefore, it is concluded that the contribution of the direct radical substitution pathway for S–S bond cleavage is more sequence and structure dependent than H-abstraction mechanisms due to its strict requirements for proper angular alignment of the reactant centers.

Additionally, hydrogen transfer from the sterically more accessible β-carbons to less exposed α-carbons is considered and the detailed discussion is provided in Fig. S15.†

## Conclusion

We report detailed experimental and theoretical studies of the mechanism of disulfide bond cleavage by a covalently attached regiospecific acetyl radical (FRIPS). Collisional activation of the model peptides derivatized by regiospecific acetyl radical conjugation yields highly selective C–S and S–S bond cleavages in both inter- and intra-peptide chain disulfide linkages. Additional collisional activations of fragments from C–S and S–S bond cleavages generate sequence information for the attached peptide chains, allowing us to locate disulfide bond linkages between specific cysteine residues. Based on DFT results, direct radical substitution at sulfur is suggested for the favored S–S bond cleavage observed in FRIPS. Using deuterium labeled model peptides, we found that both C–S and S–S bond cleavage processes can be also initiated by H-abstraction either at the α-carbons or β-carbons. Subsequent β- and γ-cleavages lead to C–S and S–S bond ruptures. We believe that gas phase fragmentation pathways discussed herein can provide insights relating to other radical-driven disulfide bond cleavages regardless of the nature of radical centers such as the benzyl^44^ and methyl pyridyl radicals^42^ and biological processes associated with disulfide bond cleavages by reactive radical species and redox stress.^64^
