# Supplementary material for: Mechanisms and energetics of free radical initiated disulfide bond cleavage in model peptides and insulin by mass spectrometry
Source: Chem Sci. 2015 May 20;6(8):4550–60. doi: 10.1039/c5sc01305d (PMC5666513; doi:10.1039/c5sc01305d)
Supplement: Supplementary file 1 [file SC-006-C5SC01305D-s001.pdf]

Electronic Supplementary Material (ESI) for Chemical Science.
This journal is © The Royal Society of Chemistry 2015

Electronic Supporting Information of

Mechanisms and Energetics of Free Radical Initiated Disulfide Bond Cleavage in Model Peptides and Insulin by Mass Spectrometry

Chang Ho Sohn[1], Jinshan Gao[1, †], Daniel A. Thomas[1], Tae-Young Kim[1, ‡], William A. Goddard III[1, 2] and J. L. Beauchamp[1,]*

[1]Division of Chemistry and Chemical Engineering, [2]Materials and Process Simulation Center, Beckman Institute California Institute of Technology, Pasadena, CA 91125, United States

Current Address: [†]Department of Chemistry and Biochemistry, and Center for Quantitative Obesity Research, Montclair State University, Montclair, NJ 07043, United States

[‡]Division of Liberal Arts & Sciences, Gwangju Institute of Science and Technology (GIST), Gwangju, 500-712, South Korea

[*]To whom correspondence should be addressed: E-mail: jlbchamp@caltech.edu

Experimental Details

Peptide Fragmentation Nomenclature

Peptide fragmentation nomenclature used in this study for c- and z-type ions produced by ECD and ETD versus a- and x-type ions produced by FRIPS is explained in Figures S1 and S2. The nomenclature of peptide fragmentation was initially proposed by Roepstorff and Fohlman[1], and Johnson et. al.[2] In this work, we follow recent notations used for electron capture dissociation spectra, where z$^{\bullet}$ ion is (z + 1) ion.

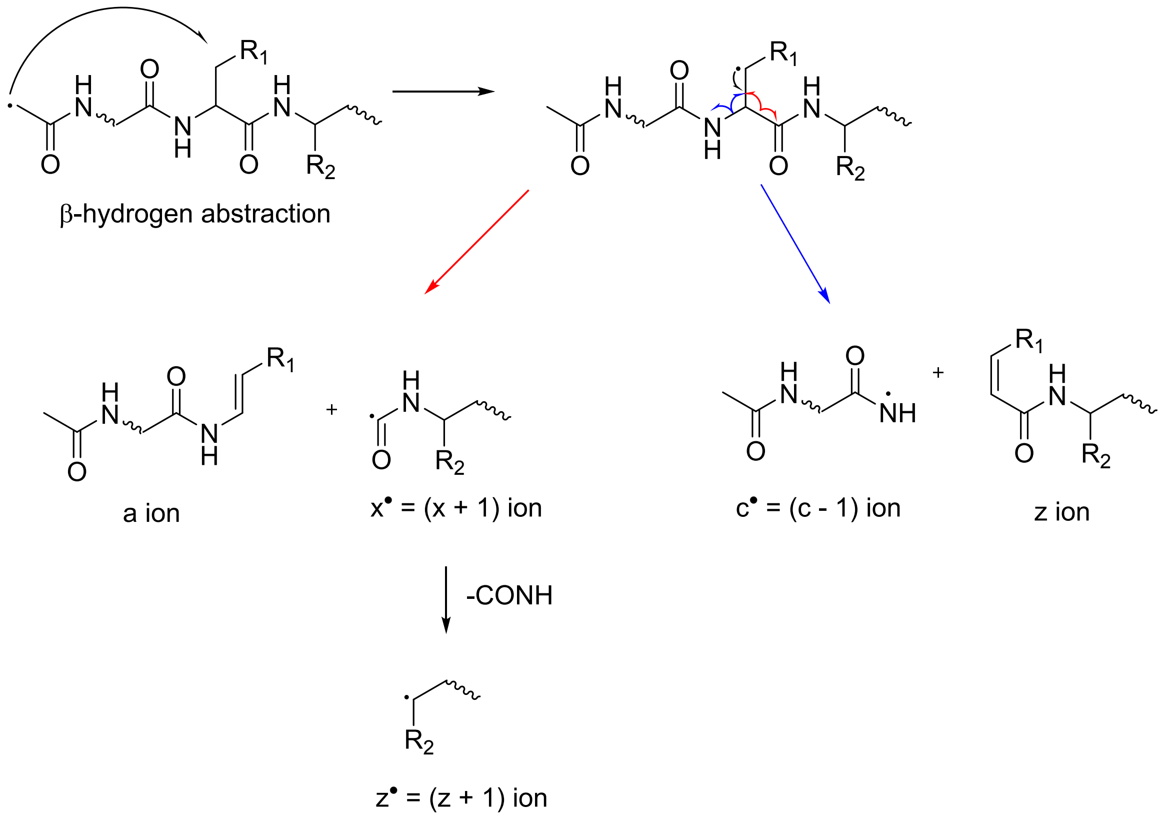

Figure S1. Peptide fragmentation by β-hydrogen abstraction followed by β-cleavage. Formation of a and z$^{\bullet}$ ions is the major pathway.

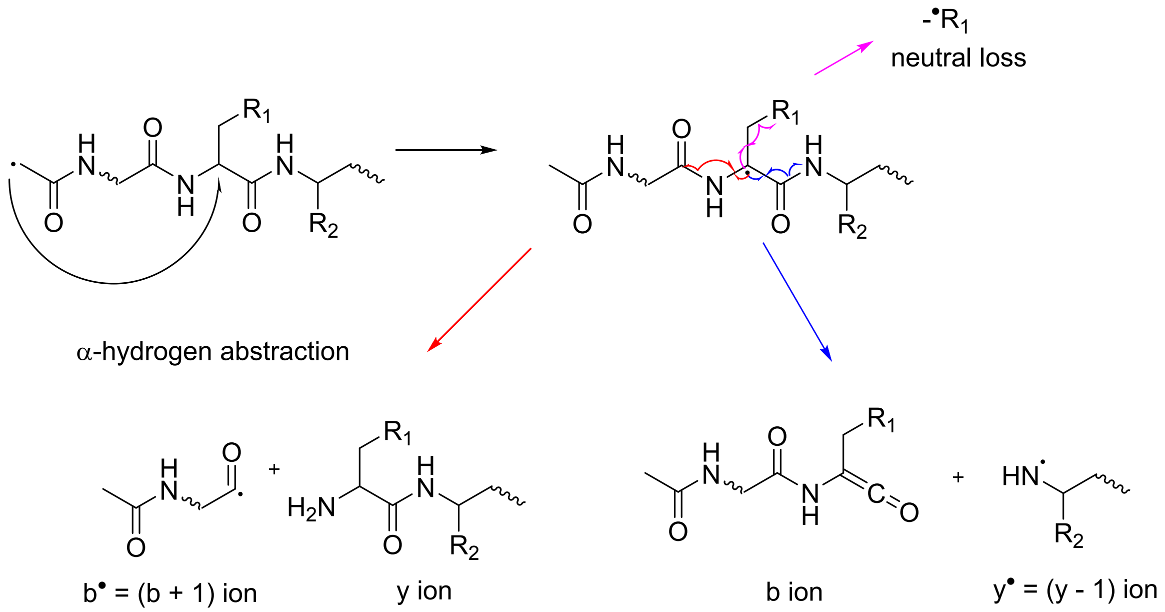

Figure S2. Peptide fragmentation by β-hydrogen abstraction followed by β-cleavage. Neutral loss is the major pathway.

Materials.

The model peptides, AARAAACAA, in 95% peptide purity, and AARAAACAA with a deuterated β-carbon at the cysteine residue, in 98% isotope purity, and 75% peptide purity, were purchased from Biomer Technology LLC (Pleasanton, CA, USA). Vasoactive intestinal peptide (VIP) from human, residue 1-12 (HSDAVFTDNYTR) that simulates tryptic peptides, and Arg8-Vasopressin (CYFQNCPRG-NH_2, Cys1 and Cys6 are connected via a disulfide bond) were purchased from AnaSpec (San Jose, CA, USA). Arg8-Conopressin G (CFIRNCPRG-NH_2, Cys1 and Cys6 are connected via a disulfide bond) was purchased from Bachem (Torrance, CA, USA). Bovine insulin was purchased from Sigma Aldrich (St. Louis, MO, USA). A soluble free radical initiator, Vazo 68, was purchased from DuPont (Wilmington, DE, USA). Acetic anhydride was purchased from Mallinckrodt Inc. (Phillpsburg, NJ, USA). All solvents are HPLC grades and were purchased from EMD Merck (Gibbstown, NJ, USA). Sequencing grade TPCK treated

trypin was purchased from Promega (Madison, WI, USA). All other chemicals were purchased from Sigma-Aldrich (St. Louis, MO, USA) unless otherwise specified. For desalting, OMIX 100 μL size C-18 tips were purchased from Varian Inc. (Palo Alto, CA, USA).

Synthesis of TEMPO-based FRIPS Reagent.

Synthesis strategy for the second generation TEMPO-based FRIPS reagent, 2,5-dioxopyrrolidin-1-yl 2-((2,2,6,6-tetramethylpiperidin-1-yl)oxy)acetate was published by Thomas *et al.*[3] The previous development by Lee *et al.*[4] was modified by replacing 2-(bromomethyl)benzoic acid methyl ester with methyl 2-bromoacetate for the current study. Briefly, TEMPO is coupled to the acetyl methyl ester group, followed by deprotection and activation of the carboxylic acid group. Ten mg/mL of the final product, 2,5-dioxopyrrolidin-1-yl 2-((2,2,6,6-tetramethylpiperidin-1-yl)oxy)acetate in acetonitrile (ACN) was reacted with 50 μg of model peptides in 100 mM phosphate buffer (pH 8.5) for 2 hr. After desalting, the resulting peptide conjugates were analyzed by electrospray ionization (ESI) mass spectrometry.

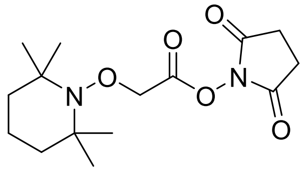

TEMPO-based FRIPS reagent

For bioconjugation of the TEMPO-based FRIPS reagent, 10 μL of bovine insulin stock 0.33 mM in DMSO (Note that bovine insulin is poorly soluble in distilled water at high concentration), 10 μL of TEMPO-FRIPS stock 10 μg/μL in acetonitrile (~32 mM, 100 eq.), and 80 μL of 10 mM phosphate buffer saline, pH adjusted to 6.3 to avoid lysine modification and disulfide scrambling,[5] were mixed and incubated at room temperature overnight with a constant vortexing at 200 rpm. The reaction mixture was desalted by HPLC using a C18 trap column (Microscience, CA) and the resulting eluent was freeze-dried by speedvac. The dried sample was

readjusted in 0.1% formic acid, 50% acetonitrile, and 50% water and directly infused into the electrospray source of the mass spectrometer.

Mass Spectrometry.

AARAAACAA and VIP peptide FRIPS experiments were performed using a LCQ Deca XP ion trap mass spectrometer (Thermo, San Jose, CA, USA). Prepared peptide solutions were directly infused to the mass spectrometer at 3 μL/min by a syringe pump with an electrospray ionization source. Critical parameters of the mass spectrometer include spraying voltage 3.5 kV, capillary voltage 41~42 V, capillary temperature 275 °C, and tube lens voltage −50~60 V. Other ion optic parameters were optimized by the auto-tune function in the LCQ tune program for maximizing the signal intensity. The precursor isolation window for MS^n experiments was set to 3.5 m/z, and normalized collisional energy in the LCQ tune program was varied from 23% to 28% based on residual precursor ion intensities. For FRIPS and CID spectra, 100 scans were recorded.

ECD and high-resolution FRIPS experiments were performed in the Proteome Exploration Laboratory of the Beckman Institute at Caltech using a 7-tesla linear ion trap Fourier transform ion cyclotron resonance (LTQ-FTICR) mass spectrometer (Thermo, San Jose, CA, USA) equipped with the Nanomate (Advion BioSciences Inc., Ithaca, NY, USA) nanospray unit. The spraying voltage was 1.4 kV, and the gas pressure was 0.3 psi. Critical parameters of the mass spectrometer include capillary voltage 49 V, capillary temperature 200 °C, and tube lens voltage 180 V. Other ion optic parameters were optimized by the auto-tune function in the LTQ tune program for maximizing the signal intensity. The target resolution at 400 m/z was set to 100,000. The precursor isolation window for ECD experiments was set to 5.0 m/z and electron irradiation occurred for 100ms at 5% of the full energy scale in the LTQ tune program, corresponding to electron energies less than approximately 1 eV. For ECD spectra, 100 scans were recorded. In

particular, the TEMPO-based FRIPS reagent conjugates of intermolecular disulfide bond containing peptides (**2**), Arg8-Conopressin G, and trypsin digested Arg8-Conopressin G were analyzed by ion trap scans in a hybrid LTQ-FTICR mass spectrometer with the nanospray and ion optics conditions described above.

An LTQ ion trap mass spectrometer (Thermo, San Jose, CA) was utilized for FRIPS analyses of insulin. The sample was infused directly to the commercial electrospray source at a flow rate of 1 μL/min. The following instrumental parameters were used: 5 kV spray voltage, 45 V capillary voltage, 125 V tube lens voltage, and 275 C° capillary temperature. The high-mass scanning mode (m/z up to 4000) was employed to monitor the singly charged insulin A-chain ion and other potential fragments.

Quantum Chemical Calculation.

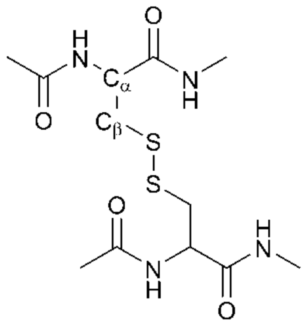

N,N'-diacetyl-cystine-*N*-methylamide

Scheme S1

N,N'-diacetyl-cystine-*N*-methylamide (Scheme S1) was used for the simple model system to describe the intermolecular disulfide bond linked peptide used in this study, CH_3CONH-[AARAAACAA]-S-S-[AARAAACAA] (**5**). Initial geometries were generated by the MC/MM conformer search using Macromol 8.0 (Schrödinger Inc., Portland, OR, USA) as implemented in Maestro 8.0 (Schrödinger Inc., Portland, OR, USA) under the linux environment. The OPLS 2005 was used for the force field model. Within 5 kcal/mol energy, all low energy conformers

were initially recorded. After manual screening of obtained structures to avoid redundancy, low energy conformers were selected for further structure optimization by density functional theory (DFT). For radical species, ad hoc assignment of the formal charge state was employed to implement MC/MM calculations for conformer searching. In particular, conformers of the C_β hydrogen abstracted *N,N′*-diacetyl-cystine-*N*-methylamide radical were searched by substitution of the β-carbon with boron to simulate the trigonal bonding environment. Each conformer was subject to a geometry optimization using Jaguar 7.5 (Schrödinger Inc., Portland, OR, USA) at the B3LYP/6-31G(d) level. By monitoring the occurrence of imaginary vibrational frequencies, only non-transition state structures (i.e. no imaginary vibrational frequency) were further optimized using a higher basis set at the B3LYP/6-311++G(d,p) level. The transition state structures were searched using the LST or QST methods by interpolating initial guesses for reactants, products and transition states. Single point energy was refined using Q-Chem 3.1 (Q-Chem Inc., Pittsburg, PA, USA) by the BMK, M05-2X, M06-2X and B3LYP density functionals with the 6-311++G(3df,3pd) basis set. The three new generation meta-hybrid functionals were chosen for their ability to more reliably predict the energetics of organic radical reactions.[6] For the open-shell systems, the spin-unrestricted method was used. All calculations were performed using computational resources kindly provided by the Material and Process simulation center at the Beckman Institute, Caltech.

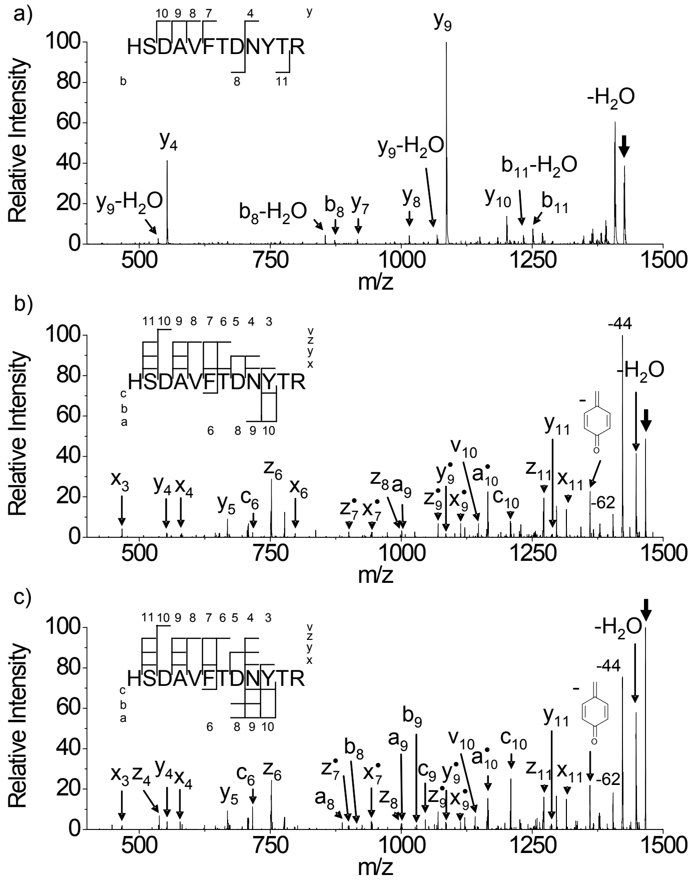

Figure S3. The CID spectrum (a) and FRIPS (MS3, Vazo 68 and the TEMPO-based reagent) spectra (b and c, respectively) of singly protonated HSDAVFTDNYTR. The CID spectrum (a) is dominated by y_4 and y_9 ions from the low energy salt bridge cleavage pathway at the C-terminal sides of aspartic acids, effected by the protonated arginine residue. This results in poor sequence coverage (6 out of 11 backbone cleavages observed, 55%). b) and c) are nearly identical, yielding mainly backbone cleavage fragments (10 out of 11 backbone cleavage observed, 91% sequence coverage) and neutral losses. Bold arrows indicate the precursor ions.

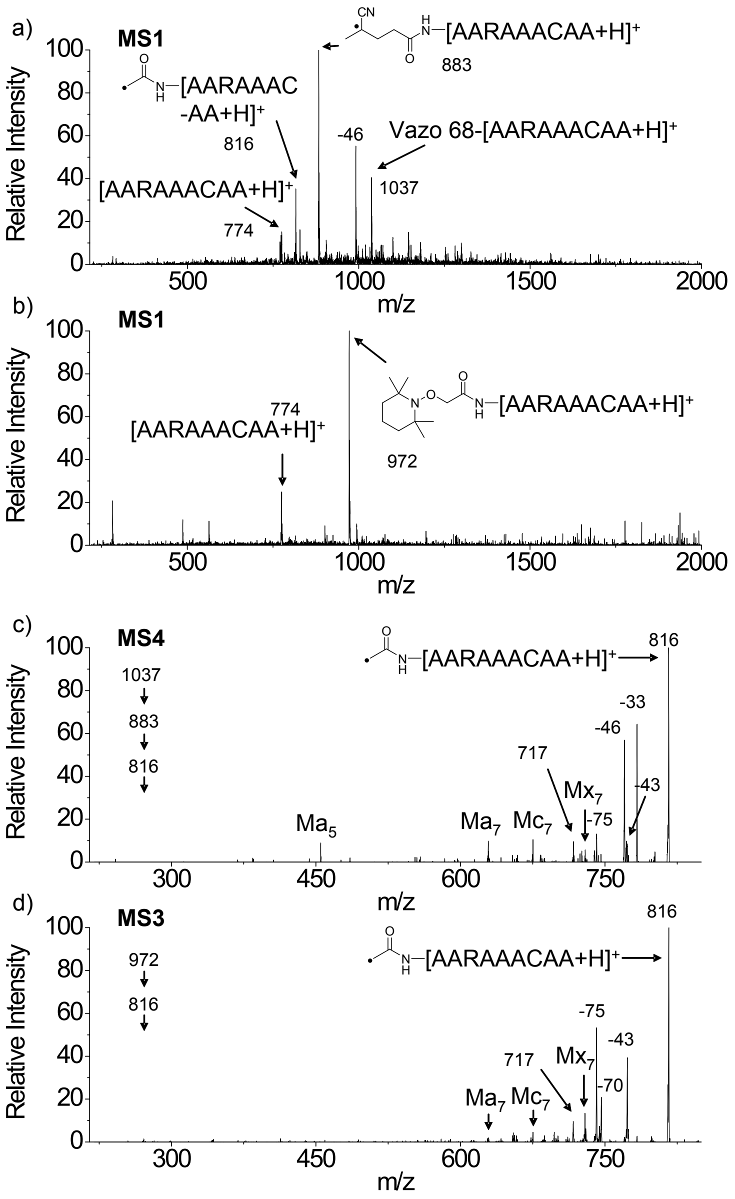

Figure S4. FRIPS of AARAAACAA. a) and b) ESI-MS1 spectra of Vazo 68 and TEMPO-based FRIPS reagent conjugated AARAAACAA peptides, respectively. c) and d) FRIPS of AARAAACAA peptides prepared by Vazo 68 and TEMPO-based FRIPS reagents. Note that the TEMPO-based FRIPS reagent requires the less number of isolation and collisional activation steps for acquisition of FRIPS spectra than that of the Vazo 68 (MS4 for Vazo 68 versus MS3 for TEMPO-based FRIPS reagent). The N-terminal peptide fragment ions that contain the acetyl group at the N-termius are marked as Ma_n, Mc_n ions, respectively.

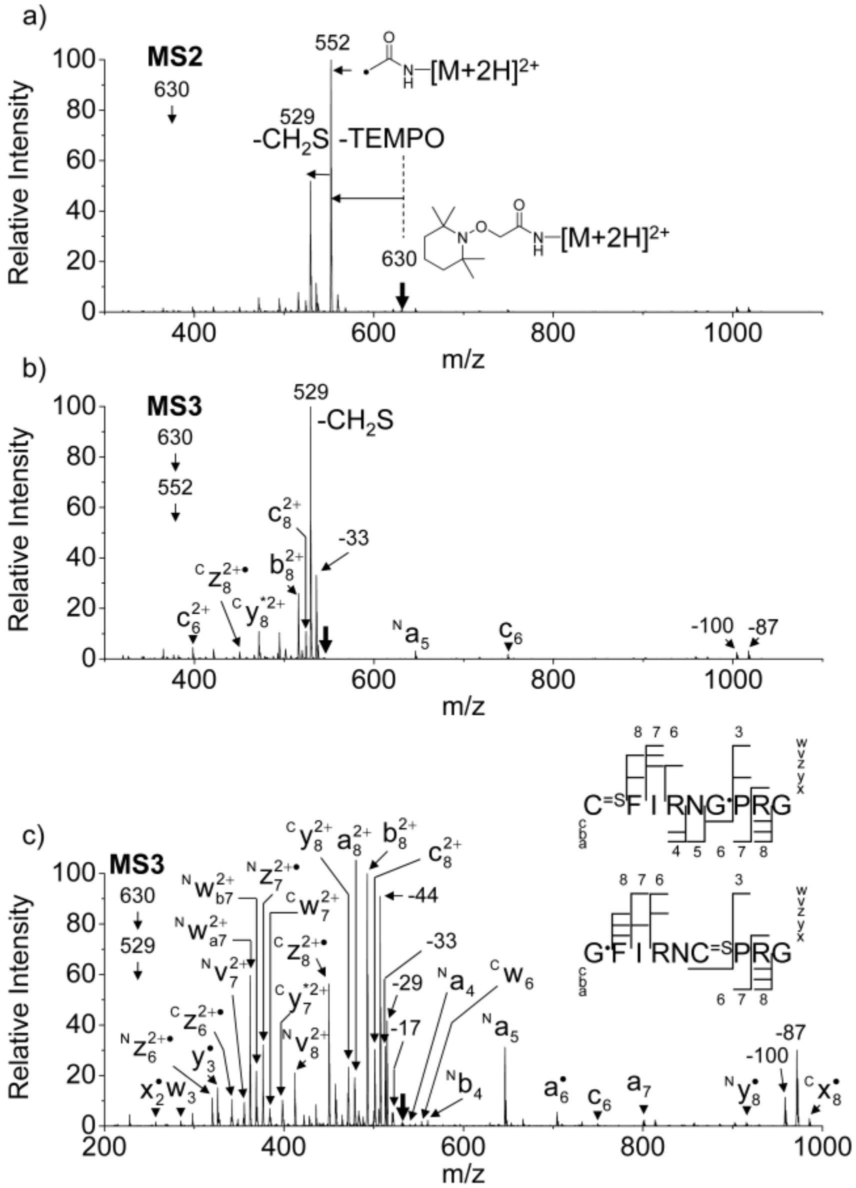

Figure S5. FRIPS of doubly protonated Arg8-Conopressin G. a) CID of doubly protonated TE¬MPO-based FRIPS conjugate of Arg8-Conopressin G at m/z 630 (MS2). b) CID of the acetyl radical containing Arg8-Conopressin G dication at m/z 552 (MS3). c) CID of the CH2S loss dication from the acetyl radical dication at m/z 529 (MS3). $C^{=S}$ could be thioaldehyde, thiomorpholin-3-one or thiirane products, G• is glycyl α-carbon radical, and "Ac-" is the acetylated N-terminal group except for the N-terminal thiomorpholin-3-one product case. Superscripts ("N" and "C") on the left side of the fragment ions indicate the position of the $C^{=S}$ residue that is located at the N-terminal or the C-terminal side of the peptide. Bold arrows indicate the precursor ions.

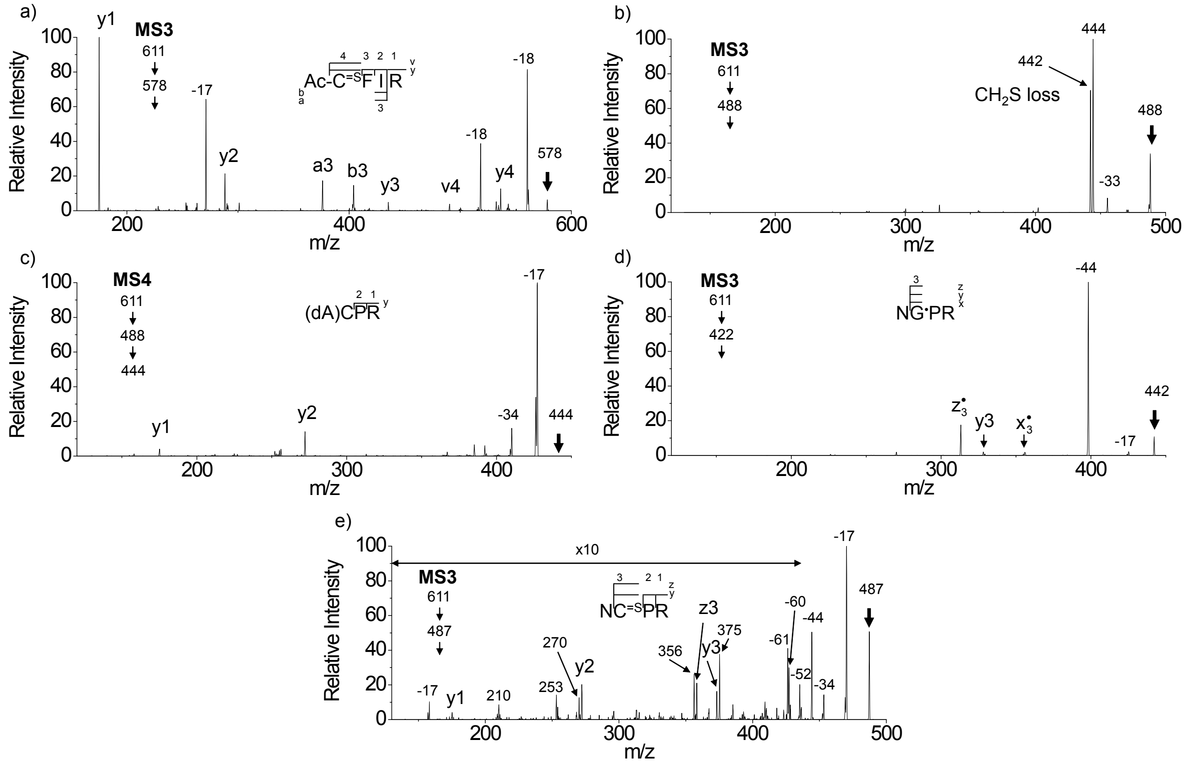

Figure S6. FRIPS of doubly protonated TEMPO-CFIR/NCPR. a) CID of the A-chain, Ac-$C^{=S}$FIR cation (even electron species) at m/z 578 (MS3), yielding the sequence of the A-chain. b) CID of the B-chain, N$C^{S\bullet}$PR cation (odd electron species) at m/z 488 (MS3). Losses of 44 (•$CONH_2$, m/z 444) and 46 (CH_2S, m/z 442) Da are prominent. c) CID of the B-chain cation at m/z 444 (even electron species, MS4). d) CID of the B-chain cation at m/z 442 (odd electron species, MS3). e) CID of the B-chain cation at m/z 487 (even electron species, MS3). The CID spectra of various B-chain peptide cations (c, d, and e) allow sequencing of the B-chain. $C^{=S}$ is thioaldehyde, thiomorpholin-3-one or thiirane products, $C^{S\bullet}$ is thiyl cysteine radical, $G^{\bullet}$ is glycyl α-carbon radical, and "Ac-" is the acetylated N-terminal group except for the N-terminal thiomorpholin-3-one product case. Bold arrows indicate the precursor ions.

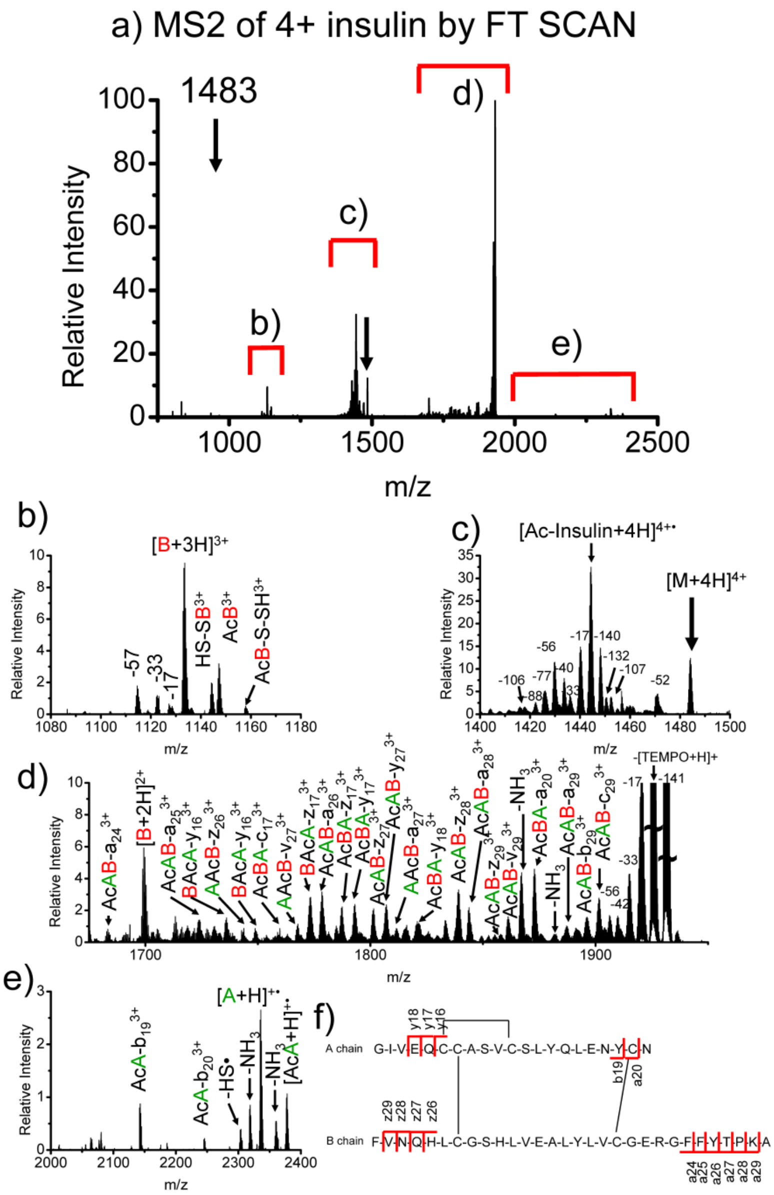

Figure S7. FRIPS of insulin 4+ ion, m/z 1483 scanned by FT ICR. a) Full spectrum scanned from m/z 500 to 2500. b) The triply charged B-chain fragments, c) TEMPO loss and other neutral losses from the precursor 4+ ion, d) abundant backbone fragments between the doubly charged B-chain fragment and the protonated TEMPO loss ion, d) the singly charged A-chain fragments, and f) insulin backbone fragmentation summary in MS2. "Ac" is an acetyl group, "A" is the A-chain fragment, and "B" is the B-chain fragment. "AcAB-a_{24}^{3+}" means the triply charged intact acetylated A-chain with a_{24} fragment of the B-chain. Most backbone fragments are observed the outside of the intermolecular disulfide bonds. Further sequencing was performed by isolating A- and B-chain fragments followed by additional collisional activation (Figure 8S). Note that the relative abundances in FT scan is biased by the charge state of the target ion so we put the ion trap spectra in the main text Figure 3 for fair comparison of disulfide bond cleavage yields with other model systems in this work (Compare the relative abundances of the singly charged A-chain fragments in FT ICR vs ion trap scans in the main text.)

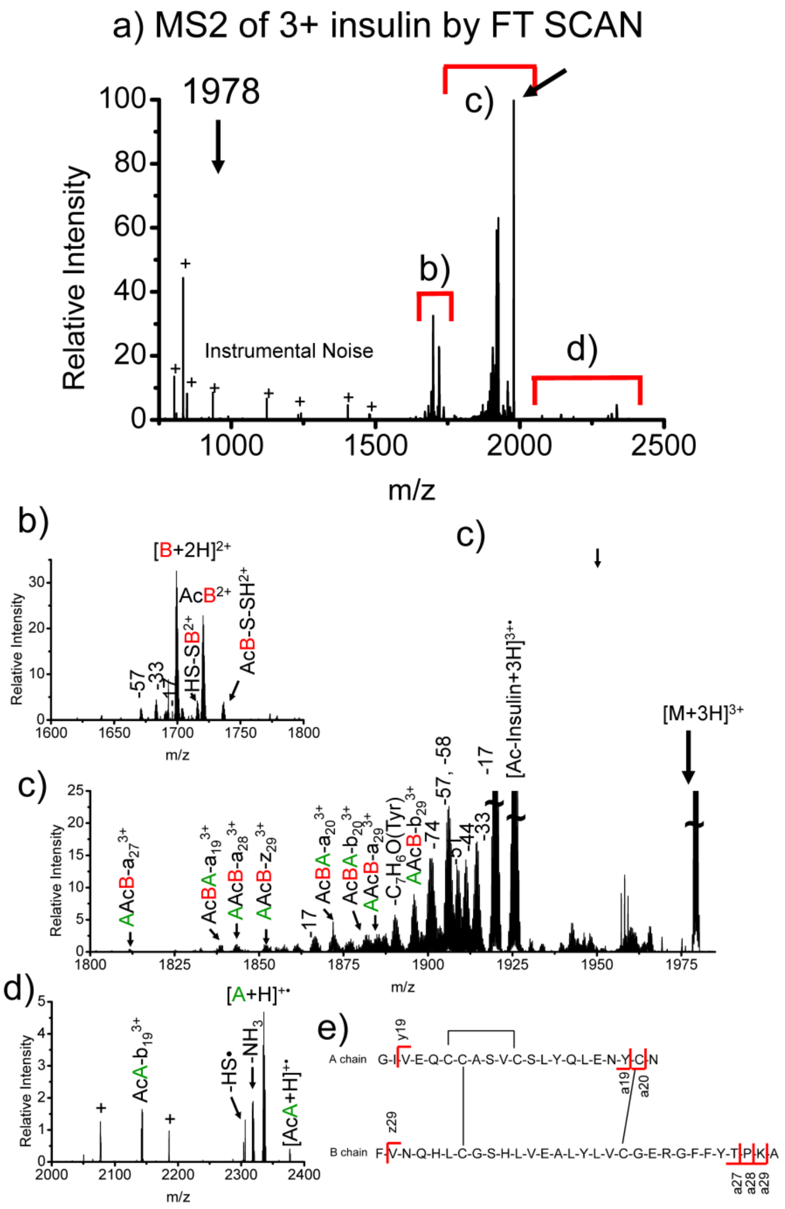

Figure S8. FRIPS of insulin 3+ ion, m/z 1978 scanned by FT ICR. a) Full spectrum scanned from m/z 500 to 2500. b) The doubly charged B-chain fragments, c) TEMPO loss and other neutral losses from the precursor 3+ ion with backbone fragments, d) the singly charged A-chain fragments, and e) insulin backbone fragmentation summary in MS2. Less backbone fragmentation was observed compared to that of the 4+ ion. As seen in the 4+ ion spectrum in Figure S6, most backbone fragments are observed the outside of the intermolecular disulfide bonds.

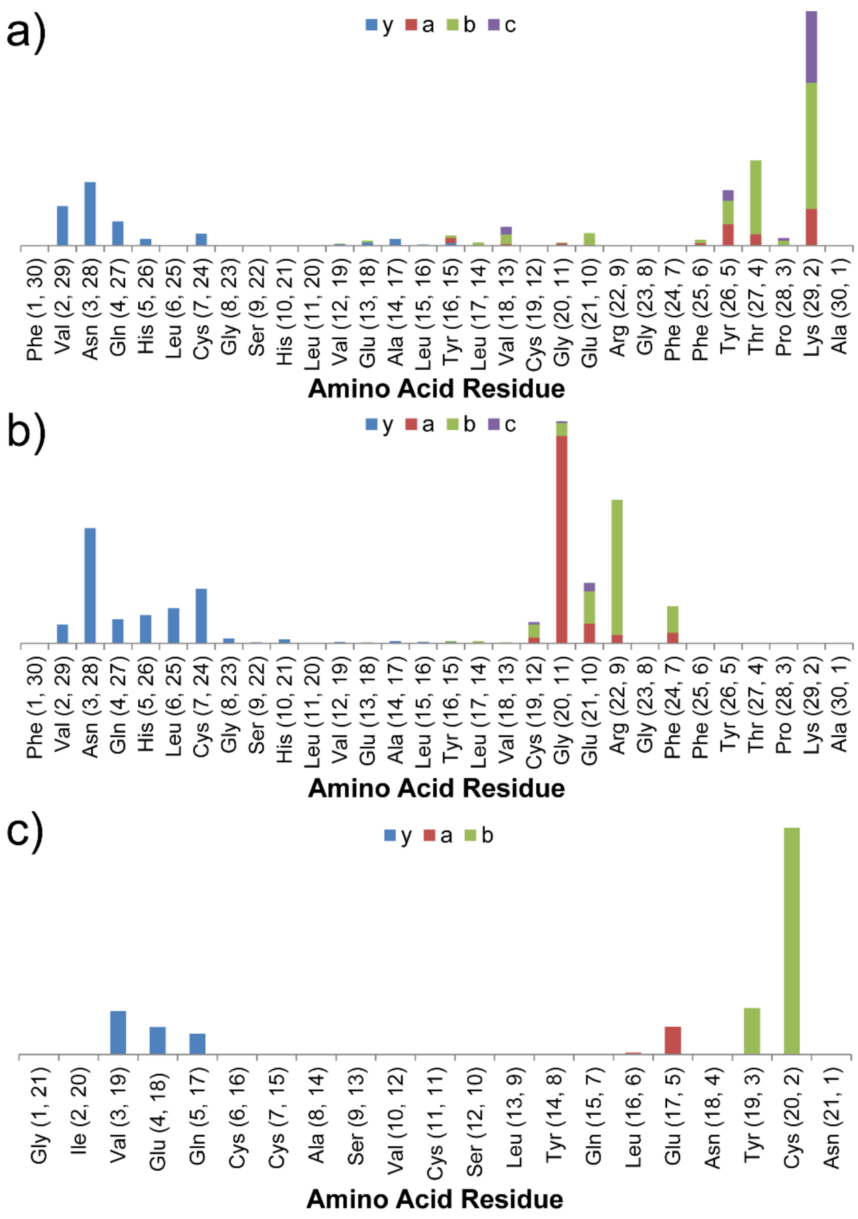

Figure S9. Insulin A and B chain sequence information obtained from MS3 following FRIPS of intact insulin. a) Relative abundances of product ions from CID of doubly charged insulin B chain ion (m/z 1699). b) Relative abundances of product ions from CID of triply charged insulin B chain ion (m/z 1134). c) Relative abundances of product ions from CID of singly charged insulin A chain ion (m/z 2335). The y-axis represents summed intensities of each ion type at a given location. Numbers in parentheses on x-axis give the residue number for a, b, c (first number) or y ions (second number).

Table S1. Product ion assignment for FRIPS of **2HH** and deviation from theoretical masses.

Peak annotation	Theoretical MS	FT MS	FT ppm	IT MS	IT ppm
B-chain CH_2S loss from $S^•$	728.3924	728.3933	1.24	728.5	85.3
B-chain $=CH_2$	741.4002	741.4020	2.43	741.5	73.3
A-chain CH_2S loss from $S^•$	770.4030	770.4052	2.86	770.4	-51.1
B-chain =S	773.3723	773.3746	2.97	773.5	106.4
B-chain $S^•$	774.3801	774.3803	0.30	774.5	96.2
B-chain $S^•$ 2nd isotope by ^{13}C	775.3835	775.3851	2.02	775.5	91.6
A-chain $=CH_2$	783.4108	783.4107	-0.18	783.5	55.9
Acetyl radical dication	794.8815	794.8803	-1.57	794.9	34.7
Acetyl radical dication 2nd isotope	795.3832	795.3826	-0.77	N/A	N/A
Acetyl radical dication 3rd isotope	795.8848	795.8855	0.94	N/A	N/A
2,2,6,6-tetramethylpiperidine loss	802.3750	802.3778	3.48	802.5	99.2
2,2,6,6-tetramethylpiperidine loss 2nd isotope	802.8767	802.8780	1.62	N/A	N/A
B-chain $SS^•$	806.3522	806.3540	2.19	806.4	14.2
A-chain =S	815.3828	815.3851	2.76	815.5	88.0
A-chain $S^•$	816.3907	816.3908	0.07	816.4	-33.1
A-chain $SS^•$	848.3627	848.3615	-1.36	848.4	1.1
A-chain $SS^•$ 2nd isotope	849.3661	849.3678	1.98	N/A	N/A
precursor ion	872.9509	872.9523	1.60	872.9	-47.9
		avg ppm	1.69		62.7

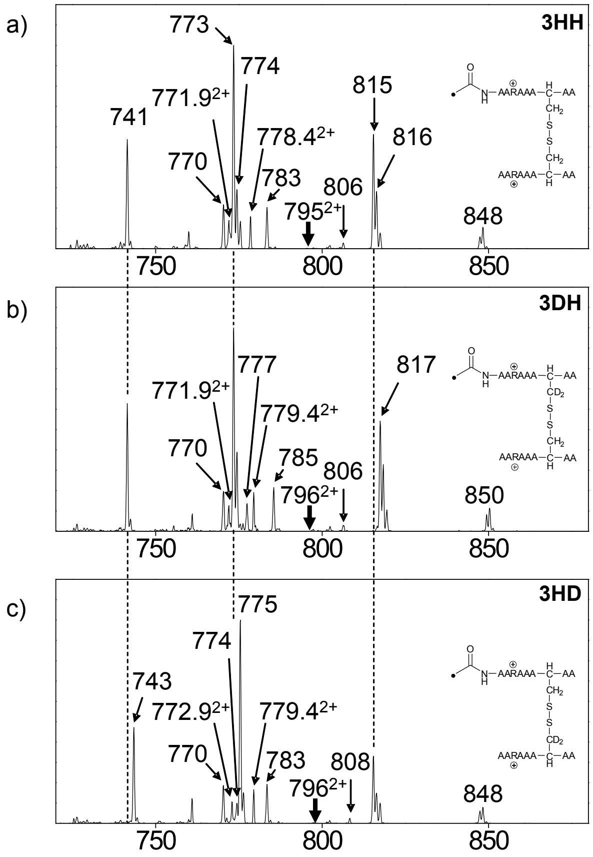

Figure S10. FRIPS MS3 (collisional activation of acetyl radical) of acetyl radical **3HH**, **3DH**, and **3HD**, (a, b, and c) respectively. Highly selective C−S (m/z 741/743, 783/785, 806/808, and 848/850) and S−S cleavage (m/z 773/775, 774/776, 815/817, 816/818) products are observed. See Figure 4c and Table 2 for the structures of the products. Almost no backbone fragmentation is observed, thus only the disulfide cleavage region is shown here. Loss of CH_2S or CD_2S from the thiyl radicals at m/z 816 (a-3HH and c-3HD) or 818 (b-3DH) yields the glycyl α-carbon radical product at m/z 770. Additional losses of $HS^•$, $HSS^•$ and CH_2S or CD_2S complicate the correlation of product ion intensities to the distribution of C−S and S−S bond cleavages solely produced by an acetyl radical.

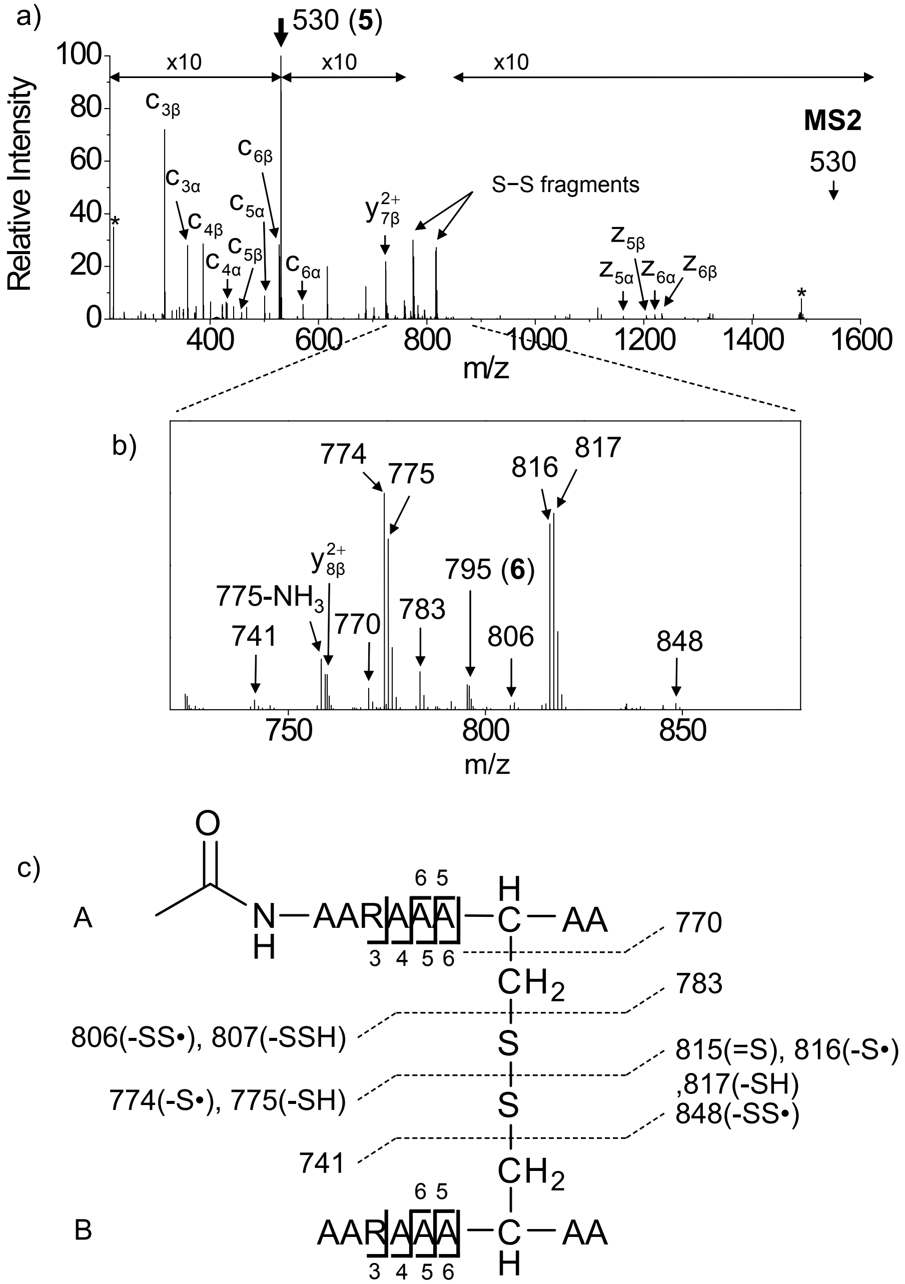

Figure S11. a) ECD of the triply charged A-chain acetylated AARAAACAA disulfide-bridged dimer (**5**, m/z 530, bold arrow). The charge reduced doubly charged Ac−[AARAAACAA]−S−S−[AARAAACAA] (**6,** m/z 795) is generated by electron capture. Several ECD backbone fragments (c and z ions) are observed. b) Expansion of the m/z range in which disulfide cleavages occur. c) Scheme showing cleavage sites and fragment m/z values from each chain.

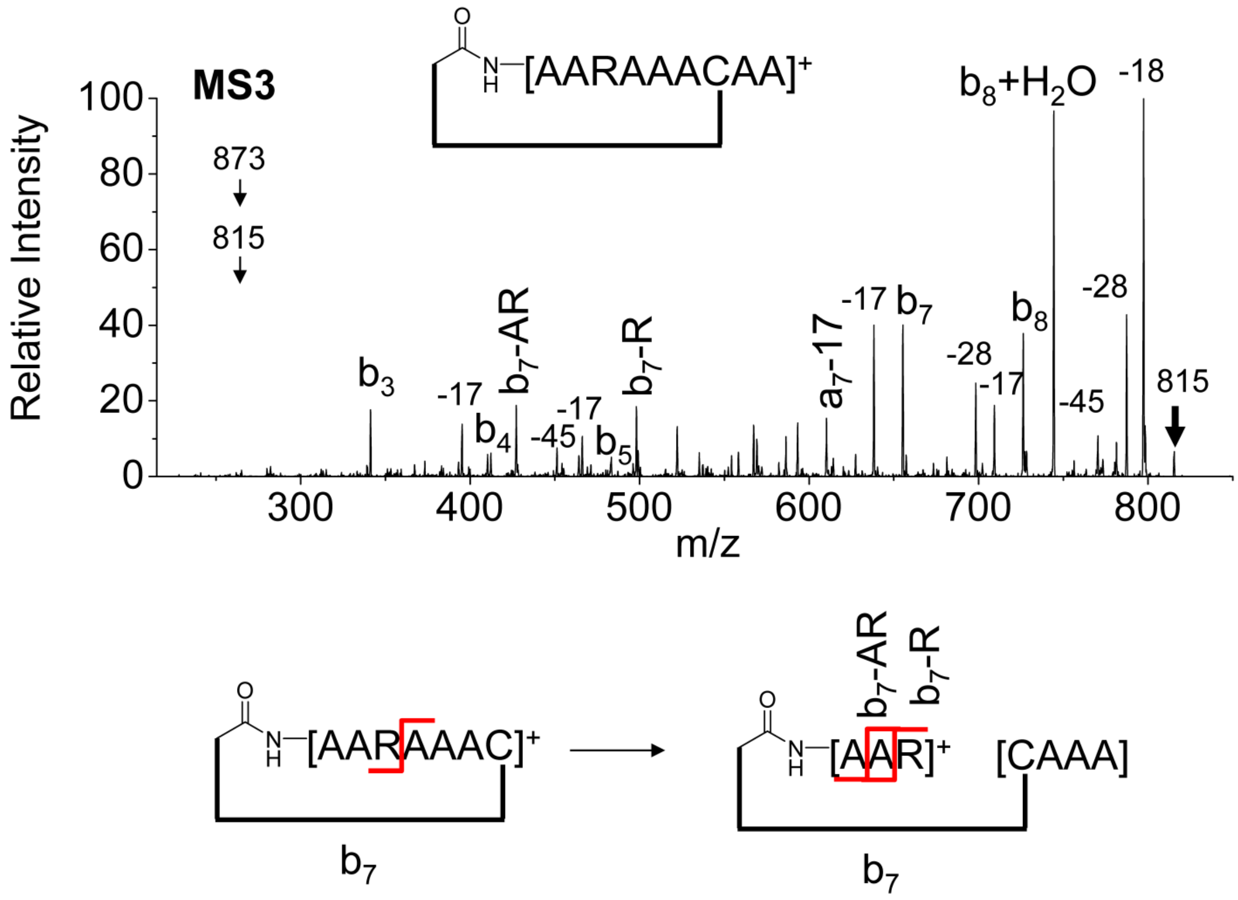

Figure S12. Collisional activation (MS3) of m/z 815 produced by disulfide bond cleavage of the AARAAACAA dimer using the FRIPS reagent. The observed product ions support a cyclic structure, with the most abundant product ions occurring at the C-terminal portion of the peptide not contained within the cyclic structure. Below the spectrum a possible mechanism for internal loss occurring within the cyclic ring is illustrated.

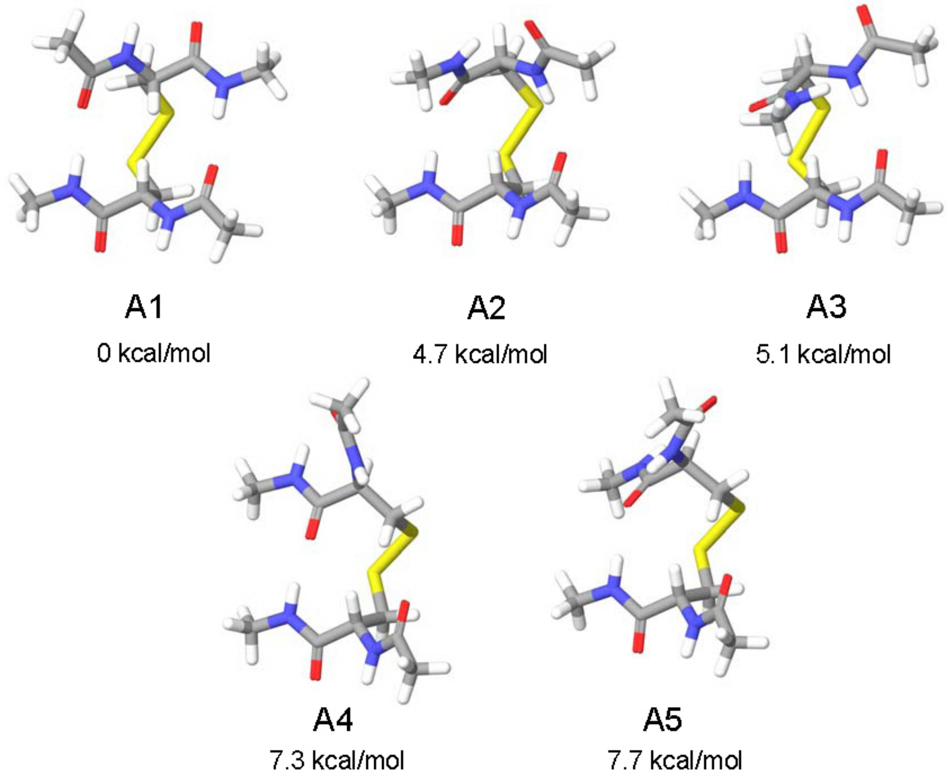

Figure S13. Relative enthalpies of low energy conformers of *N,N′*-diacetyl-cystine-*N*-methylamide at 298.15K and 1 atm. Geometry optimization and thermochemical calculation were performed using B3LYP/6-311++G(d,p) level of theory, and single point energy refinement was performed using B3LYP/6-311++G(3df,3pd) level of theory, B3LYP/6-311++G(3df,3pd)// B3LYP/6-311++G(d,p).

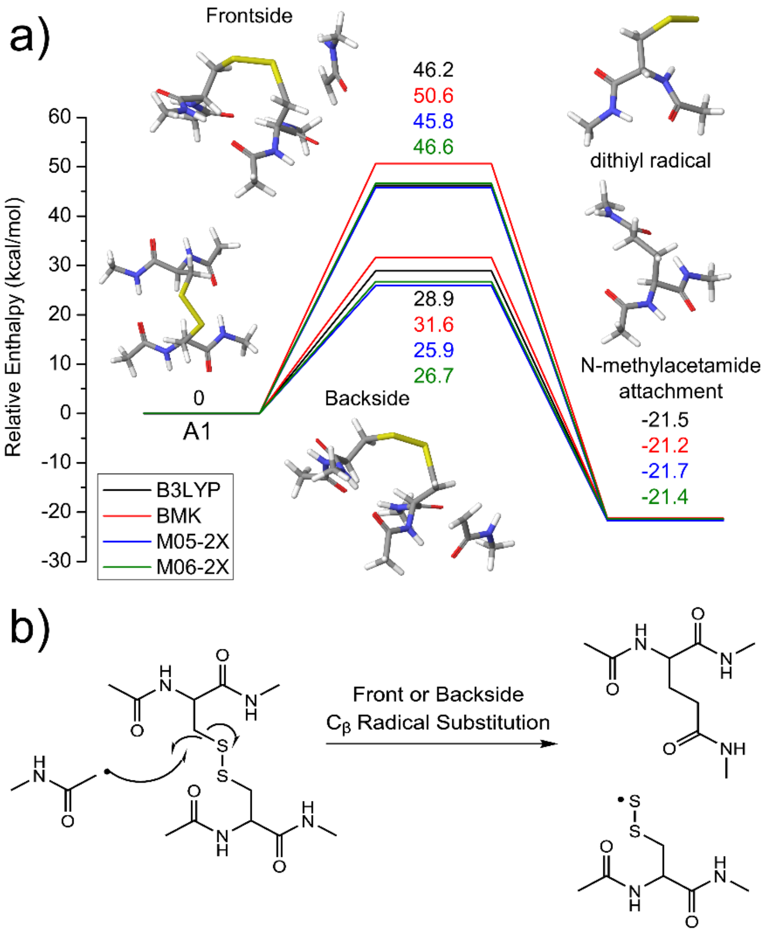

Figure S14. a) Reaction energetics for S−S bond cleavage of *N,N′*-diacetyl-cystine-*N*-methylamide by direct radical substitution at the β-carbon via front- or backside, showing relative enthalpies in kcal/mol. b) Schematic drawing of reaction mechanisms for direct radical substitution.

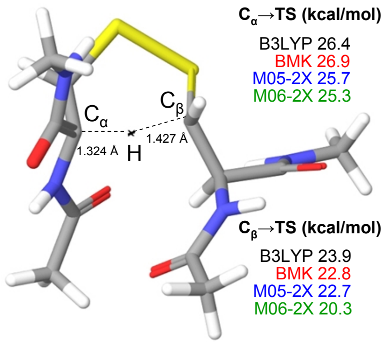

Figure S15. The optimized transition state structure of hydrogen migration between α- and β-carbons and reaction barriers from each side showing relative enthalpies in kcal/mol.

In the mechanisms involving H-abstraction, the relative barriers for H-abstraction from the α- or β-carbons determine whether C−S or S−S bond cleavage subsequently occurs. From Figure 4, it is evident that H-abstraction reactions from the α- or β-carbons have only small differences in barriers and on this basis might both be expected to occur. However, H-abstraction by the N-terminal acetyl radical group from the sterically more accessible β-carbons is likely to be favored. Therefore, the actual frequency of H-abstraction from the α-carbons may be low. To explain observed products possibly produced via the formation of the α-carbon radicals, we investigate the H-migration reaction between α- and β-carbons via a six membered ring transition state (Figure S15). If the barrier for the interchain H-migration between the α- and β-carbons is lower than that for subsequent β-cleavages of the resulting radicals generated by sterically favored H-abstraction from β-carbons, this migration can initiate various C−S or S−S bond dissociation pathways via a facile formation of the α-carbon radicals.

Figure S15 depicts the structure of the transition state for radical migration in which the transferred H-atom is approximately centered between the α- and β-carbons. The calculated reaction barrier from the β-carbon is rather high, ~20-23 kcal/mol but it is still 4-7 kcal/mol

lower than the overall endothermicity of S–S bond cleavage via the β-carbon radical. Therefore, it is energetically reasonable for the interchain H-atom migration to occur from the β-carbon to the α-carbon of a disulfide bond and the resulting α-carbon radical can undergo further C–S or S−S bond cleavages.

References

(1) Roepstorff, P.; Fohlman, J., *Biol. Mass Spectrom.* **1984,** *11* (11), 601-601.
(2) Johnson, R. S.; Martin, S. A.; Biemann, K.; Stults, J. T.; Watson, J. T., *Anal. Chem.* **1987,** *59* (21), 2621-2625.
(3) Thomas, D. A.; Sohn, C. H.; Gao, J.; Beauchamp, J. L., *J. Phys. Chem. A* **2014,** *118* (37), 8380-8392.
(4) Lee, M.; Kang, M.; Moon, B.; Oh, H. B., *Analyst* **2009,** *134* (8), 1706-1712.
(5) Chan, A. O.-Y.; Ho, C.-M.; Chong, H.-C.; Leung, Y.-C.; Huang, J.-S.; Wong, M.-K.; Che, C.-M., *J. Am. Chem. Soc.* **2012,** *134* (5), 2589-2598.
(6) (a) Boese, A. D.; Martin, J. M. L., *J. Chem. Phys.* **2004,** *121* (8), 3405-3416;(b) Zhao, Y.; Truhlar, D. G., *Theor. Chem. Account* **2008,** *120*, 215-241.
